# Advanced Nanofiber-Based Scaffolds for Achilles Tendon Regenerative Engineering

**DOI:** 10.3389/fbioe.2022.897010

**Published:** 2022-06-30

**Authors:** Senbo Zhu, Zeju He, Lichen Ji, Wei Zhang, Yu Tong, Junchao Luo, Yin Zhang, Yong Li, Xiang Meng, Qing Bi

**Affiliations:** ^1^ Center for Rehabilitation Medicine, Department of Orthopedics, Zhejiang Provincial People’s Hospital (Affiliated People’s Hospital, Hangzhou Medical College), Hangzhou, China; ^2^ Department of Orthopedics, The Second Affiliated Hospital and Yuying Children’s Hospital of Wenzhou Medical University, Wenzhou, China

**Keywords:** regenerative engineering, Achilles tendon, scaffolds, nanofibers, nanofiber technology

## Abstract

The Achilles tendon (AT) is responsible for running, jumping, and standing. The AT injuries are very common in the population. In the adult population (21–60 years), the incidence of AT injuries is approximately 2.35 per 1,000 people. It negatively impacts people’s quality of life and increases the medical burden. Due to its low cellularity and vascular deficiency, AT has a poor healing ability. Therefore, AT injury healing has attracted a lot of attention from researchers. Current AT injury treatment options cannot effectively restore the mechanical structure and function of AT, which promotes the development of AT regenerative tissue engineering. Various nanofiber-based scaffolds are currently being explored due to their structural similarity to natural tendon and their ability to promote tissue regeneration. This review discusses current methods of AT regeneration, recent advances in the fabrication and enhancement of nanofiber-based scaffolds, and the development and use of multiscale nanofiber-based scaffolds for AT regeneration.

## 1 Introduction

The AT is the largest and strongest tendon in the body, formed by the merging of the tendons of the gastrocnemius and soleus muscles (Järvinen et al., 2005). During running, the AT experiences a huge amount of strain, up to 12.5 times bodyweight. The AT’s 8% ruptures occur 2–6 cm proximal to the calcaneus, a poorly vascularized area (Hess, 2010; Uquillas et al., 2015). According to one prospective study, the AT rupture rate was 7% (Ingvar et al., 2005). Hence, the AT is the most injured tendon in the body, accounting for approximately 40% of all tendon ruptures (Veronesi et al., 2020). In the adult population (21–60 years), the incidence of AT injuries is approximately 2.35 per 1,000 people (Darrieutort-Laffite et al., 2020).

The healing and regeneration of natural tendons can be divided into three distinct stages: 1) inflammatory cells and collagen deposits migrate to the lesion site within (Ingvar et al., 2005; Grasman et al., 2015; Darrieutort-Laffite et al., 2020; Veronesi et al., 2020) days. 2) To repair damaged tendons, fibroblasts and tenocytes proliferate to generate a temporary extracellular matrix composed of type I and III collagen. 3) The final stage involves six to eight weeks of the remodeling process to form oriented type I collagen (Grasman et al., 2015; Schneider et al., 2018; Nakayama et al., 2019; Saveh-Shemshaki et al., 2019; Baiguera et al., 2020; Carnes and Pins, 2020). Natural tendon healing is usually characterized by distorted ECM and fibrous scarring, as well as functional loss resulting from dislocated collagen fibers and adhesion formation (Verdiyeva et al., 2015). In addition to conventional surgical techniques, tissue transplants have been used for acute injury with severe tendon gaps, failure of end-to-end sutures, and chronic injury. Autografts are taken from one part of the body, increasing the risk of disease at the donor site and making their use inefficient. Using allografts and xenografts to reduce donor site morbidity raises the concern of immune rejection and disease transmission (Petranto et al., 2018; Schneider et al., 2018; Li et al., 2021a; Regennass et al., 2021). As a result, the repaired tendon has weak biochemical and mechanical properties and is prone to re-injury.

The poor results of conventional treatments have prompted the development of tissue engineering technologies to produce completely integrated tendon repair tissue that has the functional and mechanical properties of natural tendons (Lim et al., 2019). Tissue engineering includes cell therapy, bioactive molecule, and biomaterial scaffolds, either used alone or in combination (Saveh-Shemshaki et al., 2019). In this regard, advanced nanofiber-based scaffolds, which can eventually mimic the mechanical and physiological properties of the Achilles tendon, have attracted a lot of attention.

This review will briefly describe the anatomy and structure of AT. Its focus is on the preparation, material, and application of advanced nanofiber-based scaffolds in tissue engineering for AT injury repair, enhancement, and regeneration.

## 2 Achilles Tendon Anatomy and Structure

The AT is the largest, thickest, and strongest tendon in the human body (Winnicki et al., 2020). It is the distal insertion of the triceps calf and consists of the soleus muscle and the two heads of the gastrocnemius muscle (lateral and medial) that are inserted into the calcaneus. Calf triceps and AT are essential structures of ankle joint plantar flexion. The gastrocnemius muscle is primarily responsible for running and jumping, while the soleus muscle is used for standing (Chiodo, 2017). The number of blood vessels around the middle tendon (exogenous supply) and in the middle of the tendon (endogenous supply) is significantly reduced, and this is also the most vulnerable part of the Achilles tendon (Chen et al., 2009).

Normal tendons are made of 20% sparse cells (mostly tendinous cells and TSPCs) and 80% dense ECM. The ECM primarily consists of a network of type I collagen fibers arranged in parallel and regular order, as well as scattered glycosaminoglycans, proteoglycans, and glycoproteins, including leucine-rich microproteoglycans (Bi et al., 2007; Sensini and Cristofolini, 2018; Millar et al., 2021). Tenocytes are tendon-specific fibroblasts, accounting for about 95% of tendon tissue. Tendinocytes are developed from tendinoblasts. Mature tendinocytes located between collagen fibrils are responsible for ECM production as well as tendon tissue maintenance and restoration (Li et al., 2021b). The TSPCs account for about 5% of tendon cells in human, rabbit, mouse, and rat tendons (Wang and Komatsu, 2016). The TSPCs have stem cell properties such as clonogenicity, pluripotent differentiation, and self-renewal. Moreover, TSPCs are mostly found in ECM protein-surrounded niche environments, and their fate is governed by the ECM-rich niche-specific components (Bi et al., 2007). From the biological point of view, autologous TSPCs may be the best option for tendon injury treatment. The ability of TSPCs to self-renewal and differentiate into tendon cells are expected to help rebuild the structure and function of damaged tendons. However, because of the limited amount of TSPCs in normal tendons, it is difficult to obtain large amounts of TSPCs from oneself (Li et al., 2021b).

**FIGURE 1:**
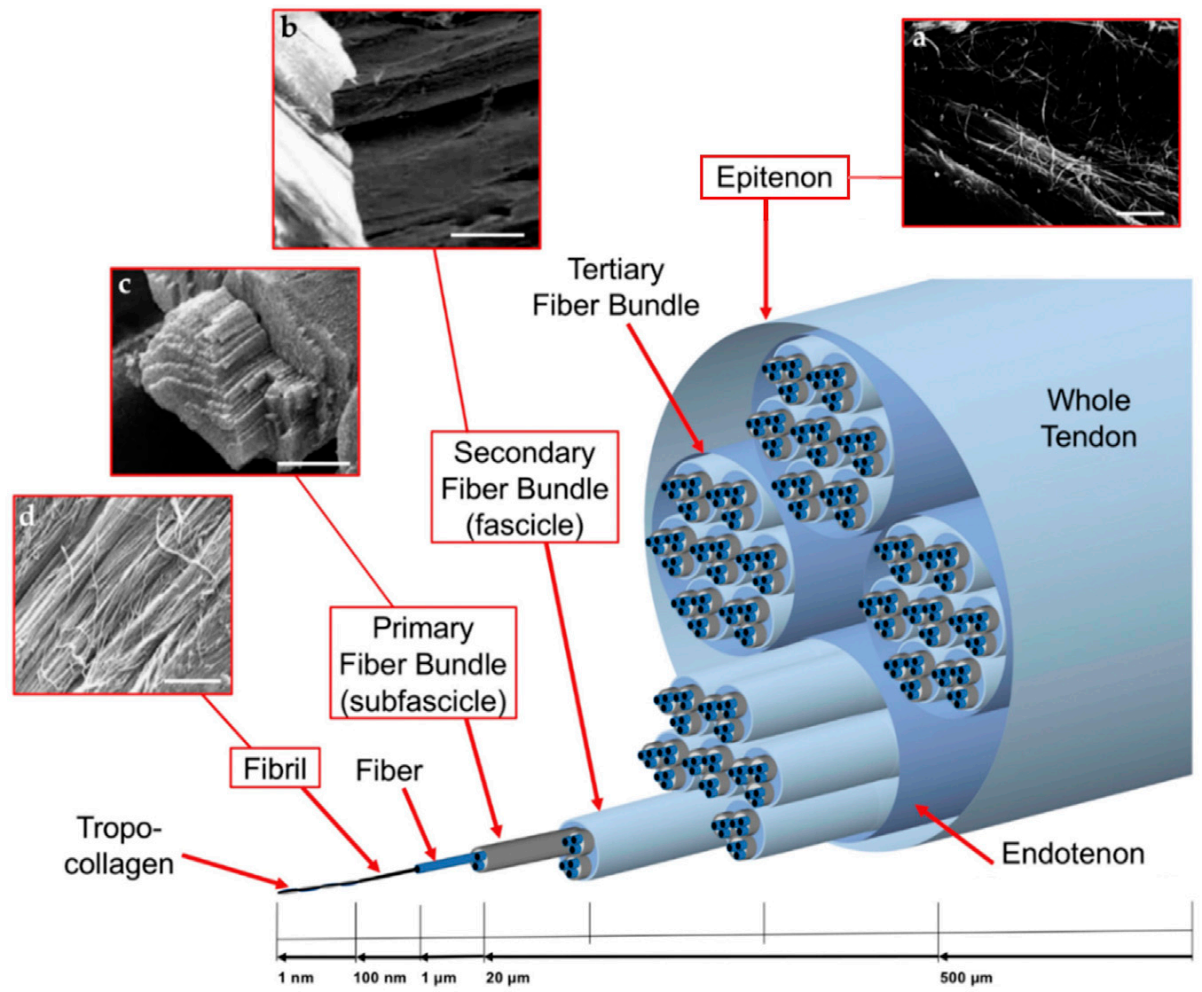
Graded arrangement of the collagen of tendons: **(A)** SEM image of epitenon fibrils (scale = 2 microns); **(B)** SEM image of secondary fiber bundle (scale = 100 micron); **(C)** SEM images of primary fiber bundle (scale = 45 microns); and **(D)** SEM image of collagen fibrils (scale = 1.8 micron). Permission to reproduce was granted under the conditions of the license (CC BY 4.0). Copyright 2018, Sensini et al.

Collagen accounts for 65%–80% of the dry mass of healthy tendons. The types I, II, III, V, and VI make up the collagens that form collagen fibrils. More than 90% of healthy Achilles tendon collagen is type I collagen. Type I collagen and its main variant type III collagen, both provide tensile strength. Type V collagen forms the core of type I collagen fibers and is involved in regulating fiber diameter. Type VI collagen is considered to connect tendinocytes to ECM molecules such as hyaluronic acid, fibrillary collagen, and core proteoglycan (Winnicki et al., 2020). Tendons consist of complex hierarchies of collagen fibers aligned axially with tendons and connected in varying degrees of aggregation (Figure 1). Collagen molecules bind together to form collagen fibrils, the smallest structural unit of tendon tissue. Fibril’s diameter ranges from 10 to 500 nanometers, depending on anatomical location, species, and age. Collagen fiber is first formed by a bunch of collagen fibrils. Then a bunch of collagen fibers forms a primary fiber bundle (sub-fascicle), and another group of sub-fascicles forms a secondary fiber bundle (fascicle). Afterward, a group of the fascicle, by turns, forms a tertiary bundle. Finally, the tertiary bundles make up the tendon, which is surrounded by the epitenon. The structures from fibers to tertiary fiber bundles are surrounded by a thin collagen membrane called endotenon, containing lymphatics, blood vessels, and nerves (Sensini and Cristofolini, 2018).

**FIGURE 2:**
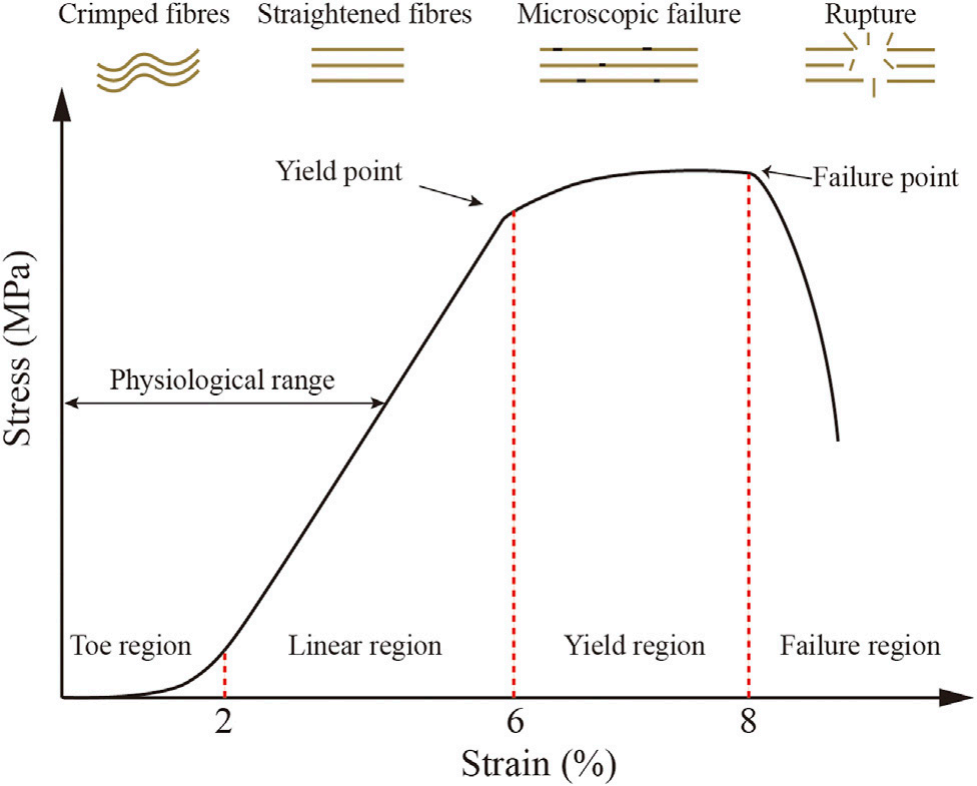
Typical tendon stress and strain curves and a schematic diagram of collagen fiber behavior. Four diverse regions may be observed. (1) Toe region (<2% strain), where fibers are crimped; (2) linear region (2%–6% strain), where fibers are straightened; (3) yield region (6%–8% strain), when microscopic failure is produced; and (4) failure region (>8% strain), where rupture is produced. (1) and (2) regions are regarded as physiological range, as previously described (Ruiz-Alonso et al., 2021).

All of these structural traits including non-linear elasticity, viscoelasticity, and anisotropy ultimately determine the specific biomechanical properties of tendon tissue (Kjaer, 2004; Ruiz-Alonso et al., 2021). The stress and strain curve formed when different stress values are applied to the tendon is referred to as non-linear elasticity. The resulting stress and strain curve has four distinct regions (Figure 2). The area I is referred to as the tendon “Toe” and shows tendon changes at less than 2% strain (low deformation). It correlates with non-destructive forces that reduce the angle of static crimp of collagen fibers without causing them to stretch further. Further stretching into the linear region (strain up to 4%), where loading causes the already-aligned fibers to stretch, the tendon exhibits reversible elastic behavior. At the end of this region lies the yield point, where some fibers begin to break slightly at 6% of the strain. Fiber breaks occur in this region unpredictably. Further elongation pushes the tendon into the rupture zone (strain > 8%), where it extends beyond its physiological limit and is completely destroyed (Maganaris et al., 2008; Morin et al., 2021). During operation, AT may be subjected to pressures up to 110 MPa, exceeding the average ultimate tensile AT stress of 100 MPa (Winnicki et al., 2020).

The enthesis is a tissue at the tendon-to-bone interface that connects two mechanically diverse materials: tendon, a submissive proteinaceous material with high toughness, and bone, a tough mineralized tissue with a higher Young’s modulus (Deymier et al., 2017). Furthermore, histology divides it longitudinally into four different regions, namely, the region of applying force. These areas are tendon (I), uncalcified fibrocartilage (II), calcified fibrocartilage (III), and bone (IV). Zone (I) consists mostly of highly aligned type I collagen. Uncalcified fibrocartilage is composed of a mixture of collagen types II and III as well as the proteoglycan aggrecan and has a much more network-like system. In the third zone, the structures are reinforced by mineral deposits at the nanoscale. The fourth area is the bone, which is mainly mineralized type I collagen (Cross et al., 2016; Rossetti et al., 2017). Under physiological load, the tensile modulus of the tendon is ∼0.4 GPa in the direction of muscle force. The tensile modulus of bones is ∼20 GPa (Hope and Saxby, 2007; Baldino et al., 2016).

The repaired tissue of a tendon injury looks like a scar and never fully recovers its pre-injury biomechanical properties (Voleti et al., 2012). The AT repair is difficult due to its low cellularity and inadequate blood supply. Hence, it has significantly promoted the development of tissue engineering. The goal of Achilles tendon tissue engineering, particularly nanoscaffolds, is to restore the original physiological and mechanical properties of the AT.

## 3 Tissue Regeneration Strategies for Achilles Tendon Injury Treatment

Three fundamental principles of tissue engineering for AT reconstruction are 1) the use of cells, 2) scaffolds (biomaterials) for transporting cells, and 3) the presence of bioactive molecules to produce cell differentiation and development (Zhang et al., 2022). To treat AT injuries, these methods can be used either in isolation or in combination (Lei et al., 2021).

### 3.1 Use of Cell Source

The limited regeneration capacity of AT is usually attributed to their low cellular content (Ruiz-Alonso et al., 2021). These cells are ultimately responsible for the synthesis of ECM components as well as the production of biologically active molecules that regulate a variety of physiological processes. Therefore, cell infiltration of the damaged area is regarded as a significant estimation for AT injury (Schneider et al., 2018). However, cell infiltration at the injured AT is not as efficient as postulated, owing to the low persistence and survival rate of cells at the original location (Geburek et al., 2016). Thus, tissue engineering is advanced in this area because cells can be integrated into scaffolds. This represents a major advantage of this technique, which allows cells to remain where damage occurs, minimizing cell migration to other tissues and possibly even detrimental effects (Niveditha et al., 2021).

Cell source is an important consideration because poor cell source selection can lead to tendon regeneration and phenotype drift toward osteogenesis, resulting in ectopic bone formation. Tenocytes and tenoblasts are two of the most commonly used cell types of tendon tissue engineering. However, due to the limited number of autologous cells available and the risk of donor site disease, getting autologous tenocytes is difficult (Wu et al., 2018). To be effectively used for clinical purposes, cells should be readily available, expandable *in vitro*, and gross in culture and implantation. They must be morally and ethically acceptable, functionally integrated with recipient tissue, and safe and non-immunogenic; that is, they must not be contaminated by any pathogen or tumorigenic (Almela et al., 2016). Stem cells are characterized as non-committable, self-renewing, long-dividing cells, which can differentiate in all cell lines. Current evidence supports that stem cells can promote regeneration during tendon healing (Migliorini et al., 2020). Since they are stem cells, it is crucial to provide them with the proper signals so that they differentiate toward tendinocyte lines, and they do not differentiate toward osteogenic lines resulting in ECM characteristics of bone tissue rather than tendon tissue (Loebel and Burdick, 2018). The most commonly used stem cells in tendon tissue engineering are MSCs (Kim et al., 2021), TSPCs (Zhang et al., 2019), and IPSCs (Tsutsumi et al., 2022), which can also give rise to tendinocytes or fibroblasts after differentiation.

The TSPCs are pluripotent adult stem cells involved in tendon healing. The TSPCs can clone, differentiate, and express specific stem cell surface markers (Bi et al., 2007). Tendon healing is poor and may deteriorate in the elderly, due to a decrease in endogenous TSPCs with age, coupled with impaired cellular activity (Yin et al., 2020). In a recent study, rat TSPCs were implanted on asymmetric CS scaffolds. *In vitro* studies revealed that TSPCs transplanted on CS scaffold showed higher levels of protein production and tenogenic-specific gene expression. At 4 and 6 weeks after implantation of all AT abnormalities in rats, the TSPCs group produced more COL1, COL3, and TNMD proteins as well as better arrangement of collagen fibers with elongated spindle cells (Chen et al., 2018). However, TSPCs account for approximately 4% of all mature tendinocytes. Given this low number, *in vitro* amplification of TSPCs is required prior to treatment to achieve therapeutic effect (Song et al., 2018; Migliorini et al., 2020). One of the major challenges is that the phenotype of TSPCs is gradually lost during *in vitro* culture, and the expression of tendinocyte-associated gene markers is significantly reduced (Zhang Y. et al., 2021).

The MSCs are pluripotent non-hematopoietic adult stem cells generated from the germinal layer of mesoderm (Pillai et al., 2017). The MSCs can be found in bone marrow, adipose, periosteum, skin, blood vessel walls, tendons, muscles, peripheral circulation, umbilical cord blood, periodontal ligament, and tooth tissue (Wang et al., 2013; Chen J. et al., 2021; El Khatib et al., 2021). A range of active molecules (e.g., BMP-12/7 and growth factor) can promote further expansion and tendinogenic differentiation (Tang and Lane, 2012; Zhou et al., 2019a; Perucca Orfei et al., 2019). The expression of tendon surface proteins (such as tendinomodulins) demonstrates a commitment to tendinogenesis. Longer dilation can induce differentiation and growth of osteoblast cell lines, therefore this stage must be strictly regulated (Chamberlain et al., 2007; Veronesi et al., 2017).

The BMSCs and ADSCs are more readily available in large quantities than TSPCs and other MSCs and have been investigated in various preclinical trials for tendon tissue healing (Leong and Sun, 2016). The BMSCs are the most often used source of stem cells for tendon tissue engineering and regeneration. Nevertheless, due to their higher proliferative capacity and easier accessibility for tissue harvesting, ADSCs are also increasingly being used to facilitate tendon regeneration, increasing the number of cells available for clinical usage (Torres-Torrillas et al., 2019). In a recent study, scaffolds loaded with and without autologous BMSCs were implanted into adult rabbit models of AT injury (Chailakhyan et al., 2021). The BMSC-loaded scaffold group developed spindle-shaped tenocytes, neovascularization, and parallel collagen fibers 6 months after surgery. The lesion area in the cell-free control group was filled with non-specific fibrotic tissue, with a small number of incompletely regenerated lesions. The rigidity of the BMSC-loaded scaffold repaired AT improved 98% of the value for the entire sample, according to biomechanical measurements. In other studies, autologous ADSC-loaded scaffolds cultured with GDF-5 were implanted in the rat AT models, and at 4 weeks after surgery showed higher cell density and more uniform COLIII distribution in the ADSC-loaded scaffolds group than the non-ADSC-loaded scaffolds groups (Aynardi et al., 2018).

Many studies have revealed that the therapeutic effect of non-tendon MSCs is mainly due to the ability of resident cells to be endowed by paracrine mechanisms or intercellular communication (Daneshmandi et al., 2020; Chen et al., 2021b; Shah et al., 2022). The MSCs regulate inflammation by secreting cytokines and stimulate AT tissue growth by secreting exosomes (Shi et al., 2019; Yu et al., 2020). Gissi et al. (2020) discovered that both high and low-dose rBMSCs-EVs could stimulate the migration of AT cells *in vitro*. They stimulated AT cell proliferation and enhanced COLI expression at higher concentrations. In the rat AT injury model, rBMSCs-EVs accelerated the remodeling phase of AT repair in a dose-dependent manner. High-dose rBMSCs-EVs resulted in better recovery of AT structure, optimal AT fiber alignment, and lower vascular density in histomorphological assessment and histology. The higher the EV concentration, the greater the expression of the COLI and the lesser the expression of the COLIII.

The IPSCs induced by mature cells under specific cell culture conditions have the potential to be used in cell therapy (Takahashi and Yamanaka, 2006; Nakajima and Ikeya, 2021). An approach based on IPSCs differentiation was used because it avoids autologous tissue harvesting and ethical concerns, as well as its high proliferative capability and minimal risk of heterotopic tissue development, this technique may be used in tissue engineering therapy (Nakajima et al., 2021; Tsutsumi et al., 2022). Komura et al. (2020) generated mouse IPSCs with tenocyte-like characteristics after tendon differentiation and facilited AT regeneration in mice.

Although tenocytes have been generated using mice MSCs or IPSCs, the use of human IPSC derived in cell therapy would be revolutionary (Czaplewski et al., 2014; Machova Urdzikova et al., 2014; Zhang et al., 2015a). Nakajima’s team has been developing an IPSC-based transplantation therapy procedure, and they recently published the first method to induce differentiation of IPSC-derived tenocytes (IPSC–tenocytes) (Nakajima et al., 2021). They recently established a novel approach to efficiently differentiate human IPSCs into tenocytes. Furthermore, IPSC–tenocyte transplantation can contribute to the recovery of motor function after AT injury in rats *via* implantation and paracrine action. The biomechanical strength of the regenerated AT was comparable to that of the healthy AT.

### 3.2 Use of the Bioactive Molecule

Bioactive molecules are molecules or compounds (including growth factors, angiogenic factors, cytokines, siRNA, DNA, hormones, and others) that are synthesized and secreted by cells, as controllers of cell proliferation, differentiation, and matrix deposition and play an important role in the development and repair of AT tissue (Raghavan et al., 2012; Ruiz-Alonso et al., 2021; Chen et al., 2022a). Bioactive compounds, particularly recombinant growth factors, are effective, readily available, dose-controlled, and stable. Specific bioactive chemical formulations can effectively induce tendon development (Zhang Y. J. et al., 2018a). Binding these bioactive molecules into scaffolds is simple. There are different approaches to this. They can be incorporated into biomaterials and then manufactured to form scaffolds with the bioactive molecules already bound or scaffolds that have been synthesized (Elsner and Zilberman, 2009). One specific approach involves preparing recombinant bioactive molecules containing scaffold material binding domain and controlling the release of the recombinant bioactive molecule by tethering the recombinant bioactive molecule to the scaffold (Sun et al., 2018).

Different types, concentrations, or release gradients of the bioactive molecule are appropriate for the proliferation and differentiation of different types of cells (Teh et al., 2015; Shiroud Heidari et al., 2021). The significant growth factors for tendon tissue engineering are IGF-1 (Olesen et al., 2021), PDGF-BB (Evrova et al., 2020), BMP-7 (Zhang P. et al., 2020), TGF-β1 (Rajpar and Barrett, 2019), VEGF (Zhou Y. L. et al., 2019; Yuan et al., 2022), BMP-12 (Rinoldi et al., 2019), SDF-1α (Chen C. et al., 2020), bFGF (Liu et al., 2013; Zhao et al., 2019), and TGF-β3 (Chen et al., 2022a). Raghavan et al. (2012) discovered that IGF-1, bFGF, and PDGF-BB increased the proliferation of all cell types (hADSCs, fibroblasts, and tenocytes) in *in vitro* scaffold experiments. The most effective proliferation stimulator among single growth factors was 50 ng/ml PDGF-BB. The ideal concentration of 50 ng/ml IGF-1, 5 ng/ml bFGF, and 50 ng/ml PDGF-BB showed the best regeneration effect of the injured tendon with numerous active molecules.

The FGF-2 belongs to the FGF family, which promotes not only cell proliferation but also angiogenesis, collagen production, and tissue remodeling (Benington et al., 2020; Zhang J. et al., 2020a). Low FGF-2 levels may be the main cause of poor tendon healing, with FGF-2 downregulated during the tendon healing process as per the studies (Zhang J. et al., 2020b). Increased FGF-2 concentration of repair site may stimulate AT regeneration (Zhang J. et al., 2020b). Guo et al. (2020) transfected FGF-2 into hTPSCs and FGF2 overexpressed hTPSCs significantly increased the expression of SCXA and COL3α1 *in vitro*. In the rat AT defect model, COLI/III expression was significantly increased in the FGF-2 group 4 weeks after surgery compared to the control group. Biomechanical tests revealed that the failure load of the FGF-2 group was higher than that of the control group 4 and 8 weeks after surgery.

Several studies have demonstrated the relevance of the combined role of the MAPK pathway and TGF-β in tenogenic differentiation (Zhang Y. J. et al., 2018a). Havis et al. (2014) performed transcriptome analysis on mouse tendinocytes isolated at various stages of development showing that TGF-β and MAPK were the two most strongly modified signaling pathways. TGF-β signaling induces the differentiation of mouse mesodermal stem cells into tendines *via* Smad2/3 *in vitro* and *in vivo*. The MAPK signaling inhibition was sufficient to activate Scx expression in mouse limb MSCs and mesodermal progenitors.

Platelet concentrates, such as PRF, PRP, and concentrated growth factor, contain high superphysiological concentrations of platelet-derived chemokines, growth factors, cytokines, and high concentrations of platelets (Chen et al., 2022b). The preparation procedure is simple and quick, and it is possible to evaluate the quality of PRP products by calculating the relative proportion of leukocytes, platelets, and growth factors. To date, the FDA has approved several commercial preparation systems for PRP and expanding, mainly in cosmetic and plastic surgery (Sharara et al., 2021), orthopedic medicine (McCarrel et al., 2014; Fang et al., 2020), sports and musculoskeletal medicine (Jiang et al., 2020), dermatology (Gentile and Garcovich, 2020), reproductive medicine (Sharara et al., 2021), and oral and maxillofacial surgery (Anitua et al., 2021). PRP can be delivered to the target by intradermal injection or topical administration. The therapy premise is that PRP injection can activate the repair mechanism and speed up tendon healing (Chen et al., 2022b). The injection in the rabbit AT injury model revealed that LP-PRP and LR-PRP improved AT recovery more than LP-PRP (Yan et al., 2017). Maghdouri-white et al. developed a novel type of TEND consisting of PDLLA and type I collagen (Maghdouri-White et al., 2021). For 2 weeks, annealed TEND rapidly adsorbs PRP and gradually releases PDGF-BB and TGF-β1. After implantation of the scaffold into a rabbit AT injury model, new AT tissue was generated, and mature, dense, regular, and directed connective tissue remodeling was observed *in vivo*.

However, a large number of randomized controlled trials have found that PRP injections do not improve objective tendon function or self-reported function or quality of life in patients with acute and chronic Achilles tendinopathy, and there is no evidence that they have any benefit on Achilles tendinopathy (de Vos et al., 2010; de Vos et al., 2011; Keene et al., 2019; Kearney et al., 2021). Because each PRP preparation method leads to different products with different biological functions, the regulation of PRP applications, both in clinical research and basic science, is confusing. Although the FDA has approved several commercial preparation systems for PRP preparation, uniform quality assessment and standardized preparation procedures have not been agreed upon worldwide (Zhang Y. J. et al., 2018b; Everts et al., 2021).

Because of the scarcity and high cost of growth factors, *in vivo* efficacy is inadequate. Chemically manufactured or naturally occurring small molecules, on the other hand, provide numerous advantages over growth factors in regulating cell behavior, including quantity control, cell permeability, cost efficiency, and time efficiency (Zhang C. et al., 2018). MLT (Yao et al., 2022), RGD (Yin et al., 2020), and simvastatin (Weng et al., 2022)-loaded nanofiber scaffolds have been proven to enhance AT regeneration *in vitro* and *in vivo*.

Although the therapeutic benefits of the bioactive molecule for tendon regeneration have been proven, there are still some challenges in using bioactive molecules, such as the potentially detrimental effects of uncontrolled release *in vivo* (de Girolamo et al., 2019).

### 3.3 Use of Biomaterials Scaffolds

The scaffold’s primary role in AT tissue creation is to offer biomechanical support, a bionic matrix for cell proliferation, differentiation, and migration, and to promote AT integration and growth (Paredes and Andarawis-Puri, 2016). Scaffolds are developed and created to mimic the shape and function of natural tissue ECM and to generate ideal cell interactions (adhesion, differentiation, and proliferation) that contribute to the formation of new functional tissues (Lin et al., 2018; Ruiz-Alonso et al., 2021). Several scaffold manufacturing approaches have been reported over the past decade, including collagen matrix–based creation (Dewle et al., 2020; Uday Chandrika et al., 2021), particle leaching (Ye et al., 2018), rapid prototyping (Billiet et al., 2012; Gu et al., 2016), solvent casting (Yeo et al., 2015), freeze drying (Shahbazarab et al., 2018), and fibrous structures (Tiwari et al., 2016; Reid et al., 2021; Uribe-Gomez et al., 2021). Sun et al. (2018) used a rat model SDF-1α loaded collagen scaffolds manufactured from bovine type I and type III collagen for AT regeneration. The findings showed that collagen scaffolds can enhance the diameter of collagen fiber, facilitate the expression of type I collagen of AT, and improve the mechanical characteristics of regenerated AT 4 and 12 weeks after surgery. Li et al. (2019a) implanted a parallel PCL microfiber bundles template subcutaneously in rats, resulting in collagen-rich ECM deposition and cellularization in the template. The polymer template and cells were then removed to form autologous ECM scaffolds with aligned and hollow microchannel structures. Three months after surgery, the mechanical strength of the regenerated AT filled with tenocyte was comparable to that of the pre-injury state AT.

Natural organizational microarchitecture is widely acknowledged as being important in functional organizational behavior. Cells in natural tendons also align along the direction of collagen fibers. This cell arrangement stimulates the formation of collagen matrix (e.g., COLI and COLIII) and key tendinins proteins (Wu Y. et al., 2017). Therefore, the use of fibrous scaffolds in tissue engineering is beneficial to its microstructure, which is similar to that of ECM. Aligned fibrous scaffolds, in particular, can be used to guide cell alignment and create tissues with anisotropic microstructure (Wu et al., 2019; Nagiah et al., 2021). The tendon regeneration fibrous structures can be divided into three types: nanofiber structure, microfiber structure, and nano/microfiber structure. The nanoscale properties of the nanofibers lead to effective cell attachment, proliferation, and differentiation in nano/microfibrous scaffolds, whereas coarser fibers in micron diameters can be chosen for mechanical strength (Yongcong et al., 2019; O'Brien et al., 2016; Tan et al., 2021). The aligned nanofibers are postulated to be suitable for providing topographic cues for tendon seed cells to guide cell-oriented growth and enhance cell proliferation and differentiation to generate functional tendon tissue. The anisotropic structure also enhances the tensile strength of the scaffold while responding to directional stimuli such as uniaxial mechanical loads. Therefore, the directional fiber terrain scaffold is expected to provide appropriate biophysical cues to help cells elongate along the direction of the directional fiber, thereby increasing cell proliferation rate and function (Chen et al., 2021c; Sarıkaya and Gümüşderelioğlu, 2021).

The insertion can be separated into four discrete but structurally continuous zones within a few hundred microns of the tendine-bone insertion location. The collagen fibers are parallel and aligned in the tendon region. Before adhering to the bone, the tendon fibers break into thinner, smoother interface fibers and spread symmetrically along the insertione (Lei et al., 2021). The implanted tissue had varied mineral content, collagen orientation, and protein type gradients at the interface at the micron scale (Calejo et al., 2019). Even after surgical repair, the injured tendon-bone interface does not regenerate the well-aligned collagen fibers found at natural attachments during healing. The healed scar tissue is an order of magnitude weaker than natural tissue and does not integrate well into the bone (Lei et al., 2021; Shiroud Heidari et al., 2021; Zhu et al., 2021). Therefore, an enhanced tendon-to-bone repair can be achieved by a combination of strategies: I) inducing the establishment of a grading interface; II) promoting bone formation; and III) promoting deposition of aligned collagen, all of which will result in higher adhesion strength and better integration (Zhu et al., 2021). Typical anisotropic scaffolds typically consist of three zones: aligned fibers, random fibers, and interfaces. These three zones are morphologically distinct but structurally continuous, resembling changes in the direction of collagen fibers at the tendon-bone insertion site.

## 4 Nanofiber-Based Scaffolds

### 4.1 Technology of Preparing Nanofiber-Based Scaffolds

Nanofiber-based scaffolds are prepared using various techniques. The best-known nanofiber manufacturing techniques include molecular self-assembly, phase separation, and electrospinning (Eskandari et al., 2017; Manoukian et al., 2017; Ko, 2020).

**FIGURE 3:**
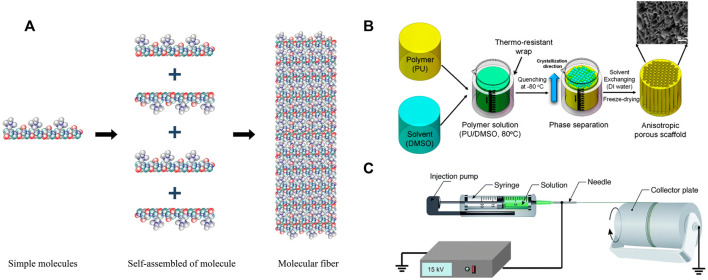
Schematization representation of the nanofiber manufacturing technology. **(A)** Self-assembly. Permission to reproduce was granted under the conditions of the license (CC BY 4.0). Copyright 2019, Nemati et al. (2019). **(B)** Thermal phase separation for the preparation of anisotropic polyurethane porous nano-scaffolds. Permission to reproduce was granted under the conditions of the license (CC BY 4.0). Copyright 2018, Zeinali et al. (2021). **(C)** Electrospinning. Permission to reproduce was granted under the conditions of the license (CC BY 4.0). Copyright 2019, Nemati et al. (2019).

Self-assembly often involves intermolecular bonding (Barnes et al., 2007). Using non-covalent forces such as electrostatic, van der Waals forces, hydrogen bonds, hydrophobicity, and π–π stacking interactions, simple molecules and macromolecules assemble rapidly into stable, organized, and well-defined structures (Lyu et al., 2020) (Figure 3A). These bonds are often weak, but when they combine to form a single unit during assembly, they affect the structural stability and conformation of the assembly and have a significant impact on the interactions between supramolecular structures and other tissues, cells, and molecules (Nemati et al., 2019; Yao et al., 2020). Natural biopolymer-based materials, such as proteins, DNA, and polysaccharides, offer excellent alternatives to tissue-engineered scaffolds. Due to a wide range of non-covalent interactions (ionic, hydrogen bonding, and hydrophobicity) between molecular chains, they exhibit superior self-assembly capabilities (Zhang R. et al., 2021). Since the late 1980s, interest in peptides derived from natural proteins with self-assembly properties has increased. Consequently, numerous engineered peptides that mimic the conformation of self-assembled peptides of protein molecules have been developed (Chen and Zou, 2019; Park, 2020). In response to external stimuli, secondary structures such as α-helices and β-folds are responsible for assembly. The pH, temperature, and ionic strength are the most critical factors in supramolecular nanofiber formation (Koutsopoulos, 2016; Vedhanayagam et al., 2017; Delfi et al., 2021). Polysaccharides such as cellulose, chitosan, and chitin have highly similar structures and are rich in hydrogen bonds, which makes it easy for them to form nanofibers through parallel self-assembly (Zhang R. et al., 2021). By adding molecules to nanofibers *via* step by step self-assembly, thin and dense functional molecular layers are formed on the nanofibers’ surface, resulting in the formation of multifunctional nanomaterials (Ma et al., 2021).

The phase separation process can be initiated by a non-solvent or by heat, and it has been widely used to make foams or porous membranes for separation and filtration methods (Asadian et al., 2020). Typically, non-solvent-induced phase separation results in a matrix with an uneven pore structure, which is undesirable for tissue scaffolds, which require a homogeneous pore structure (Yao et al., 2020; Asadian et al., 2020). Thermal-induced phase separation occurs when a homogeneous multi-component system (polymer, filler, solvent, drug, etc.) becomes thermodynamically unstable under specific conditions, resulting in the formation of two distinct phases, a polymer-poor phase and a polymer-rich phase (Figure 3B). After solvent removal by sublimation, extraction, or evaporation, the polymer phase is transformed into the skeleton of the porous scaffold, while the removed solvent is responsible for the final porosity (Zeinali et al., 2021). Changing formulation variables, such as solvent polymer concentration and mixture composition, can significantly affect the morphology of the resultant scaffold (Sabzi et al., 2021).

Electrospinning is a simple and widely used technique for producing nanofibers for application in tissue engineering (Han et al., 2021; Sensini et al., 2021a; Song et al., 2021). The electrospinning device is so simple that it can be used in almost any laboratory. The primary components include an injection pump, spinneret (usually a hypodermic needle with a blunt tip), conductive collector, and high-voltage power supply (direct current or alternating current) (Xue et al., 2019; Xue et al., 2017). In electrospinning, a high electric field is applied to the polymeric solution drawn using a capillary, and the jet begins when the electrostatic charge exceeds the surface tension (Figure 3C). Finally, by stretching the droplets of the polymer solution, the surface charge density increases as the solvent evaporates, and the fine fibers are collected on a grounded collector (Sundaramurthi et al., 2012; Sundaramurthi et al., 2013; Jayasree et al., 2019; Zhang C. et al., 2020).

Changes can be made to the polymer, its concentration in a liquid solution, polymer molecular weight, feed rate, and electric field voltage to change the diameter, composition, and direction of the nanofiber (Kalantari et al., 2019; Wu et al., 2020a).

#### 4.1.1 Comparison Between Different Nanofiber Production Technologies

The nanofiber structures formed by self-assembly of polymers are significantly thinner than nanoscaffolds prepared by electrostatic spinning. Self-assembled nanofibers, however, have several disadvantages, including low productivity, complex processing, and relatively high cost. Furthermore, self-assembled nanofibers have poor mechanical properties when compared to those prepared using other technologies (Nemati et al., 2019; Ren et al., 2021; Eygeris et al., 2022). Although the phase separation method has advantages such as low cost, ease of manufacture, and simplicity, it also has disadvantages, including lab-scale nanofiber production, long processing time, nanofiber porosity, difficulty to control structural instability, and limitation to polymers suitable for phase separation processes (Dahlin et al., 2011; Kang et al., 2020).

Electrospinning nanofibers have a more porous structure and a greater surface-to-volume ratio, providing more surface area for cell attachment. The high specific surface area allows easier oxygen penetration and prevents fluid accumulation (Kumbar et al., 2008; Moshiri et al., 2013; Jayasree et al., 2019; Nemati et al., 2019). One of the important properties of electrospun nanofibers is the ability to combine different types of materials by electrospinning. In addition to the ability to use a variety of materials, one of the main advantages of electrospinning nanofibers for tendon tissue engineering is the capacity to control the orientation of nanofibers. Moreover, the electrostatic spinning process allows the fabrication of aligned and random nanofiber structures to simulate tendon alignment (Dewle et al., 2020; Mao et al., 2020; Sensini et al., 2021b; Zhao et al., 2021).

### 4.2 Nanofibre-based Scaffolds Material

AT reconstruction (Sankar et al., 2017) employs natural polymers such as silk fibroin protein (Yi et al., 2018), collagen (Sandri et al., 2016), chitosan (Chen et al., 2018), sodium alginate, and gelatin, as well as degradable synthetic polymers such as PCL (Fotticchia et al., 2018), PLA (Wu et al., 2020b), PLGA (Hasmad et al., 2018), PET (Cai et al., 2018a), and PEO (Table1). Different materials used as scaffolds for tendon tissue engineering have their advantages and disadvantages (Chen et al., 2021a). The advantage of synthetic polymer scaffold polymers is that their sizes may be easily modified to meet requirements and they degrade slowly. In general, the use of synthetic polymer scaffolds constrained by the adverse effects of their degradation products *in vivo* (such as inflammatory reactions) and their relatively poor integration with host tissues (Chen et al., 2021a). PGA, PLA, and PLGA are considered biocompatible and cause only mild or minor foreign body reactions because their hydrolysis degradation product (glycolate lactate) is typically present in the metabolic pathway of the human body (Bianchi et al., 2021). However, their extensive degradation occasionally causes local inflammation due to the accumulation of acidic degradation products that are difficult to eliminate (Hafeez et al., 2021). PCL is also biocompatible and degrades significantly more slowly than PGA, PLA, and PLGA, making it a desirable material for long-term scaffolds such as tendons (Silva et al., 2020; Zhu et al., 2021). Because of its excellent biocompatibility and biodegradability, the synthetic polymer PLGA is a copolymer used in the majority of FDA-approved therapeutic devices. PLGA significantly decreased peritendinous adhesions, an effect that may be associated with histopathological findings of inflammation, vascular density, and fibrosis (Russo et al., 2020; Uyanik et al., 2022).

**TABLE 1:** Summary of nanofiber-based tendon regeneration scaffolds.

References	Application	Animal model	Scaffold	Material	Cell	Bioactive molecule	Morphology		Mechanical properties						
							Fiber diameter	Porosity (%)		Young’s modulus (MPa)	Ultimate stress (MPa)	Yield stress (MPa)	Ultimate stress (%)	Stiffness (N/mm)	Maximum force (N)
Yang et al. (2019)	Achilles tendon	*In vivo* rat	Aligned and Random	Insoluble collagen	rBMSCs	—	2–4 μm		As-fabricated	Radial tensile: 31.12 ± 1.19 (CMA) 43.28 ± 2.49 (CMR) axial tensile: 79.19 ± 9.39 (CMA) 74.91 ± 1.23 (CMR) 138.33 ± 15.84 (normal) 41.56 ± 9.51 (repair) 34.52 ± 8.19 (CMA) 10.51 ± 1.42 (CMR) 8.14 ± 1.74 (defect)		Radial tensile: 8.97 ± 0.78 (CMA) 11.97 ± 0.58 (CMR) radial tensile: 18.45 ± 0.91 (CMA) 13.81 ± 0.39 (CMR)	Radial tensile: 45.68 ± 5.80 (CMA) 36.37 ± 3.29 (CMR) radial tensile: 28.33 ± 0.81 (CMA) 25.85 ± 2.36 (CMR) 23.26 ± 2.63 (normal) 24.80 ± 2.19 (repair) 31.71 ± 4.43 (CMA) 34.69 ± 0.86 (CMR) 47.19 ± 2.13 (defect)		
									Transplant after 8 weeks		22.45 ± 1.80 (normal) 5.32 ± 1.05 (repair) 5.02 ± 0.18 (CMA) 1.92 ± 0.17 (CMR) 2.23 ± 0.20 (defect)			37.90 ± 3.26 (normal) 25.09 ± 4.55 (repair) 24.57 ± 3.95 (CMA) 12.05 ± 2.60 (CMR) 9.64 ± 0.64 (defect)	70.78 ± 9.01 (normal) 45.02 ± 2.50 (repair) 43.69 ± 7.51 (CMA) 26.46 ± 4.62 (CMR) 32.38 ± 7.72 (defect)
Fernandez ‐ Yague et al. (2021)	Achilles tendon	*In vivo* rat	Aligned	PVDF-TrFE	—	—	690 ± 110 nm (non-piezoelectric with drawing) 540 ± 120 nm (piezoelectric w/o drawing) 513 ± 80 nm (piezoelectric with drawing)	—	As-fabricated	14.5 ± 1.7 (non-piezoelectric with drawing) 56.6 ± 7.6 (piezoelectric w/o drawing) 61.8 ± 8.1 (piezoelectric with drawing)	16 ± 0.3 (non-piezoelectric with drawing) 15 ± 3.7 (piezoelectric w/o drawing) 31 ± 4.2 (piezoelectric with drawing)	—	91.3 ± 10.4 (non-piezoelectric with drawing) 46.4 ± 6.1 (piezoelectric w/o drawing) 39.4 ± 3.2 (piezoelectric with drawing)	—	—
Yin et al. (2015a)	Achilles tendon	*In vivo* rat	Aligned and Random	PLLA	rBMSCs	—	1068 ± 190 nm (aligned) 739 ± 129 nm (random)	—	Transplant after 8 weeks	24.42 ± 2.20 (aligned) 20.86 ± 3.56 (random)	—	∼6.5 (aligned) ∼6 (random)	—	∼32 (aligned) ∼29 (random)	87.12 ± 5.61 (aligned) 77.09 ± 13.27 (random)
Wang et al. (2016)	Achilles tendon	*In vivo* rat	Aligned and random	PCL/PEO/gelatin	hDFs	—	1074 ± 158 nm (aligned) 416 ± 114 nm (random)	—	As-fabricated Scaffolds of seeded Cell After 12 weeks transplant subcutaneously 12 weeks transplant after 12 weeks	0.53 ± 0.059 (aligned) 0.42 ± 0.065 (random) 3.07 ± 0.67 (aligned) 0.76 ± 0.17 (random) 1.74 ± 0.45 (aligned) 0.59 ± 0.17 (random) ∼6 (control) ∼16 (aligned) ∼15 (random)	134.91 ± 15.94 (aligned) 83.32 ± 11.15 (random) 154.39 ± 24.44 (aligned) 81.80 ± 16.07 (random) 78.74 ± 6.59 (aligned) 51.77 ± 14.51 (random) 261.54 ± 23.48 (control) 206.41 ± 52.59 (aligned) 200.76 ± 49.14 (random)	—	—	—	9.53 ± 1.13 (aligned) 5.89 ± 0.79 (random) 10.91 ± 1.73 (aligned) 5.78 ± 1.14 (random) 5.56 ± 0.47 (aligned) 3.66 ± 1.02 (random) ∼10 (control) ∼25 (aligned) ∼25 (random)
Yao et al. (2022)	Achilles tendon	*In vivo* rat	Patch-shaped nanofibrous scaffolds	PCL/ALG	rTPSCs	MLT	∼200 nm nanofibrous	—	As-fabricated	1.50 ± 0.17 (PCL/MLT electrospun membrane) 0.38 ± 0.03 (PCL/MLT-ALG scaffolds)	—	—	—	—	—
Chen et al. (2017)	Achilles tendon	*In vivo* rabbit	Aligned and Random	PCL/SF	rDFBs	—	549.4 ± 172.4 nm (RP) 582.5 ± 178.6 nm (RPSF) 363.7 ± 116.0 nm (APSF)	—	As-fabricated transplant after 12 weeks	15.28 ± 5.31 (RP) 3.93 ± 0.05 (RPSF) 70.52 ± 2.83 (APSF)	1.48 ± 0.21 (RP) 0.06 ± 0.01 (RPSF) 0.94 ± 0.11 (APSF)	39.75 ± 6.61 (RP) 2.05 ± 0.30 (RPSF) 1.72 ± 0.19 (APSF)	—	29.9 ± 5.8 (normal) 4.5 ± 0.7 (acellular RPSF) 10.1 ± 1.8 (acellular APSF) 8.5 ± 1.1 (cells/RPSF) 18.0 ± 3.8 (cells/APSF)	382.3 ± 58.2 (normal) 114.3 ± 11.6 (acellular RPSF) 216.4 ± 33.0 (acellular APSF) 167.3 ± 25.4 (cells/RPSF) 310.9 ± 73.5 (cells/APSF)
Wang et al. (2021)	Achilles tendon	*In vivo* rat	Parallel-aligned	Collagen	rTSPCs	rPOSTN		—	As-fabricated transplant after 12 weeks	11.15 ± 1.08 (ACF) 85.05 ± 6.03 (normal) 51.71 ± 8.01 (ACF-rP) ∼30 (ACF) ∼10 (defect)	—	10.60 ± 0.65 ACF ∼23(Normal) ∼12 (ACF-rP) ∼6 (ACF) ∼2 (Defect)	—	—	33.31 ± 2.06 ACF ∼90 (normal) 59.55 ± 8.09 (ACF-rP) 44.32 ± 4.32 (ACF) 27.22 ± 7.56 (defect)
Chen et al. (2021a)	Achilles tendon	*In vivo* rat	Aligned and random	SF	hPDLSCs	—	—	—	As-fabricated	∼70 KPa random ∼60 KPa aligned	—	—	—	—	∼1.1 random ∼1.0 aligned
Zhang et al. (2018c)	Achilles tendon	*In vivo* rat	Aligned and random	PLLA	mTSPCs	TSA	1.63 ± 0.38 μm (A-TSA) 1.73 ± 0.37 μm (A) 1.44 ± 0.23 μm (R-TSA) 1.51 ± 0.23 μm (R)	—	As-fabricated transplant after 4 weeks	513.09 ± 64.02 (A-TSA) 51.59 ± 11.68 (R-TSA) 51.23 ± 24.95 (A-TSA) 31.00 ± 9.42 (R)	—	12.50 ± 1.06 (A-TSA) 2.70 ± 0.32 (R-TSA) 6.65 ± 1.38 (A-TSA) 4.20 ± 0.98 (R)	—	44.50 ± 5.32 (normal) 28.65 ± 10.01 (A-TSA) 19.44 ± 5.38 (R)	38.27 ± 5.92 (A-TSA) 31.20 ± 5.63 (R)
Yuan et al. (2021)	Achilles tendon	*In vivo* rat	Aligned core-shell	COL1-CS (shell)/PLLA (core)	—	—	1.60 ± 0.16 μm (PLLA/CS-COL1) 1.91 ± 0.23 μm (PLLA)	—	As-fabricated	805.63 ± 31.47 (PLLA/CS-COL1) 1973.37 ± 36.85 (PLLA)	—	30.57 ± 1.94 (PLLA/CS-COL1) 39.83 ± 3.04 (PLLA)	122.97 ± 4.23% (PLLA/CS-COL1) 72.9 ± 62.95% (PLLA)	—	—
Maghdouri-White et al. (2021)	Achilles tendon	*In vivo* rat/Rabbit/cadaver	Aligned	PDLLA/Collagen I	Human tenocytes/hMSCs	PRP	∼500 nm	—	As-fabricated scaffolds of seeded Cell After7 days	152.2 ± 15.85 ∼160 (PRP Coated) ∼30 (Non-coated)	—	13.6 ± 0.26 ∼25 (PRP Coated) ∼8 (Non-coated)	∼20 (PRP Coated) ∼30 (Non-coated)		8.4 ± 1.77 ∼10 (PRP Coated) ∼12 (Non-coated)
Weng et al. (2022)	Achilles tendon	*In vivo* rat	Aligned	PLGA	—	Simvastatin	330.6 ± 137.3 nm (1:1) 390.3 ± 134.4 nm (2:1)	—	Transplant after 1 week transplant after 2 weeks transplant after 4 weeks	—	—	—	—	—	14.96 ± 2.76 (normal) 12.43 ± 2.36 (PLGA/S) 2.59 ± 1.89 (PLGA) 3.06 ± 4.79 (control) 11.01 ± 1.06 (normal) 16.47 ± 8.09 (PLGA/S) 9.48 ± 1.65 (PLGA) 4.95 ± 1.70 (control) 26.14 ± 4.13 (normal) 13.20 ± 3.70 (PLGA/S) 9.22 ± 2.35 (PLGA) 11.82 ± 3.09 (control)
Yin et al. (2020)	Achilles tendon	*In vitro*	Nanofibers	RADA	hTSPCs	RGD	10 nm	—	—	—	—	—	—	—	—
Almeida et al. (2019)	Achilles tendon	*In vivo* rabbit	Dimensional hybrid	Bovine bollagen molecule	—	—	272 ± 183 nm (aligned nanofibers) 2.54 ± 0.68 μm (polymerized microfibers)	randomly formed: 36.12% ± 6.59%; highly aligned: 11.01% ± 3.97%	As fabricated	43.81 ± 4.19 kpa (cross-linked) 4.05 ± 1.28 kpa (non-cross-linked)	—	2.69 ± 0.47 (cross-linked) 0.40 ± 0.11 (non-cross-linked)	61.34 ± 4.71 (cross-linked) 98.55 ± 7.23 (non-cross-linked)	—	28.33 ± 2.19 (cross-linked) 4.56 ± 1.92 (non-cross-linked)
Moshiri et al. (2013)	Achilles tendon	*In vivo* rabbit	Sheath and core	Collagen/PDS	—	—	—	17.62% ± 4.88%	As fabricated: transplant after 60 days after 120 days	4.05 ± 1.28 kpa (collagen) 43.81 ± 4.19 kpa (cross-linked) 610.46 ± 56.92 kpa (PDS: cross-linked) 0.24 ± 0.09 (control) 0.62 ± 0.14 (collagen) 0.87 ± 0.17 (collagen-PDS) 0.95 ± 0.15 (control) 1.99 ± 0.23 (collagen) 2.13 ± 0.26 (collagen-PDS)	0.40 ± 0.11 (Collagen) 2.69 ± 0.47 (cross-linked) 9.08 ± 1.79 (PDS: cross-linked) 5.81 ± 0.99 (control) 9.84 ± 2.24 (collagen) 13.19 ± 1.66 (collagen-PDS) 19.62 ± 1.39 (control) 26.71 ± 2.32 (collagen) 26.88 ± 2.92(collagen-PDS)	0.40 ± 0.11 (collagen) 2.69 ± 0.47 (cross-linked) 9.08 ± 1.79 (PDS: cross-linked)	98.55 ± 7.23 (collagen) 61.34 ± 4.71 (cross-linked) 14.81 ± 1.28 (PDS: cross-linked) 20.45 ± 2.3 (control) 13.82 ± 0.91 (collagen) 12.84 ± 0.71 (collagen-PDS) 17.38 ± 2.33 (control) 10.99 ± 1.05 (collagen) 9.91 ± 0.83 (collagen-PDS)	3.39 ± 0.54 (control) 24.37 ± 2.03 (collagen) 31.87 ± 3.8 (collagen-PDS) 11.7 ± 2.01 (control) 64.91 ± 5.06 (collagen) 78.15 ± 4.42 (collagen-PDS)	10.82 ± 3.91 (control) 63.72 ± 2.99 (collagen) 81.42 ± 7.84 (collagen-PDS) 31.42 ± 5.77 (control) 129.9 ± 16.07 (collagen) 152.9 ± 8.15 (collagen-PDS)
Chen et al. (2017)	Achilles tendon	*In vivo* rabbit	Sheath and core	Collagen type I/PDS	—	Bovine platelet gel (BPG)	—	—	—	—	—	—	—	—	—
Cai et al. (2018b)	Transition/achilles tendon	*In vivo* rat	Dual-layer aligned and random	SF/P (LLA-CL)	—	—	445 ± 180 nm (aligned layer) 486 ± 142 nm (random layer)	—	Transplant after 6 weeks transplant after 12 weeks	—	—	—	—	9.9 ± 1.9 (ARS) 9.3 ± 1.4 (RS) 5.8 ± 1.3 (control]) 21.5 ± 3.5 (ARS) 15.6 ± 1.6 (RS) 10.0 ± 1.1 (control)	43.9 ± 7.5 (ARS) 41.4 ± 5.7 (RS) 25.3 ± 5.9 (control]) 83.2 ± 12.4 (ARS) 66.2 ± 6.6 (RS) 50.6 ± 3.5 (control)
Madhurakkat Perikamana et al. (2018)	Transition/tendon	*In vivo* rat	Aligned and random	PLLA/ PD	ADSCs	PDGF-BB	∼600 nm				—				
He et al. (2021)	Transition/tendon	*In vitro*	Aligned and random	PCL)/HA/ZnO	MC3T-E1/ATDC5/Mouse primary fibrochondrocyte/Mouse primary tenocytes	—	—	—	—	—	—	—	—	—	—
Haoxuan Li et al. (2020)	Transition/tendon	*In vitro*	Electrospinning	PLGA	—	—	428 ± 124 nm	—	As fabricated	—	—	33.6 ± 8.2 (pristine PLGA) 56.1 ± 10.1 (welded PLGA)	—	—	—
Guner et al. (2020)	Tendon	*In vitro*	Dual core-shell nanofiber scaffolds	PCL/Gelatin	hADSCs	GDF-5	5.925 ± 3.23 μm (PCL) 1.715 ± 0.7 μm (PCL/gelatin)	76.7 ± 9.8 (FSPCL) 63.1 ± 5.3 (FSPCL/ESPCL) 66 ± 3.4 (FSPCL/ESPCLGel) 79.3 ± 4.4 (ESPCL) 81.4 ± 5.7 (ESPCL Gel)	As-fabricated	∼30.8 (FSPCL) ∼34.5 (FSPCL/ESPCL) ∼33.5 (FSPCL/ESPCLGel 3:1)	∼32 (FSPCL) ∼41 (FSPCL/ESPCL) ∼41 (FSPCL/ESPCLGel 3:1)	∼23.5 (FSPCL) ∼26.5 (FSPCL/ESPCL) ∼27.5 (FSPCL/ESPCLGel 3:1)	—	—	—
Wu et al. (2021)	Tendon	*In vitro*	wavy nanofibrous scaffolds	PPDO/SF	hADMSCs/hTCs	—	526 ± 227 nm (PPDO WNSs) 531 ± 224 nm (4/1 PPDO/SF WNSs) 591 ± 233 nm (2/1 PPDO/SF WNSs)	—	As-fabricated	15 ± 4 (PPDO WNSs) 28 ± 7 (4/1 PPDO/SF WNSs) 43 ± 6 (2/1 PPDO/SF WNSs)	31 ± 1 (PPDO WNSs) 25 ± 1 (4/1 PPDO/SF WNSs) 17 ± 1 (2/1 PPDO/SF WNSs)	—	—	—	—
Yang et al. (2022)	Transition/tendon	*In vivo* rat	Electrospinning	PLA/ZIF-11/HKUST-1	—	—	∼1μm	—	As-fabricated	42.14 ± 3.7 (PLA-H/Z) 15.8 ± 2.5 (PLA) 9.2 ± 0.9 (PLA-Z) 11.4 ± 1.8 (PLA-H)	2059.0 ± 14.5 KPa (PLA-H/Z) 867.5 ± 113.3 KPa (PLA) 555.9 ± 61.8 KPa (PLA-Z) 824.9 ± 52.4 KPa (PLA-H)	—	—	—	—
Russo et al. (2020)	Tendon	*In vitro*	Aligned and Random	PLGA	oAECs	—	2.5 ± 0.27 μm aligned 2.1 ± 0.19 μm random	—	As-fabricated	—	26 ± 1.75 aligned 15 ± 0.87 random	—	344 ± 24.89 aligned 240 ± 12.34 random	—	—
Chen et al. (2021c)	Tendon	*In vitro*	Aligned and random sheath	PCL /HA (core)	Tenocytes of rabbits	PRP (core)	410 ± 96 nm (random) 483 ± 116 nm (random + core) 362 ± 138 nm (aligned + core)	83.1 ± 5.6 (random) 86.8 ± 1.8 (random + HA/PRP core) 69.4 ± 3.6 (aligned + HA/PRP core)	As-fabricated	8.90 ± 0.88 (random) 18.64 ± 4.08 (random + core) 76.02 ± 9.21 (aligned + core)	2.21 ± 0.99 (random) 1.61 ± 0.51 (random + core) 6.62 ± 0.21 (aligned + core)	—	31.3 ± 1.4 (random) 23.63 ± 1.1 (random + core) 45.65 ± 1.0 (aligned + core)	—	—
Ramos et al. (2019)	Tendon	*In vitro*	Electrospinning	PCL/CA	hUMSCs	Insulin	732 ± 335 nm (PCL) 886 ± 426 nm (PCL + 25% CA) 724 ± 350 nm (PCL + 50% CA) 474 ± 305 nm (PCL + 75% CA)	—	As-fabricated	—	—	—	—	—	10.79 ± 1.49 (PCL) 6.53 ± .48 (PCL + 25% CA) 5.33 ± .82 (PCL + 50% CA) 2.90 ± 0.42 (PCL + 75% CA)
Sarıkaya and Gümüşderelioğlu, (2021)	Tendon	*In vitro*	Aligned	SF/P3HB	rAdMSCs	GDF-5	699 ± 203 nm	—	As-fabricated Scaffolds of seeded Cell after 21 days of culture	197 ± 8 (SF/P3HB) 182 ± 17 (S-SF/P3HB) 56.8 ± 1.5 (A-SF/P3HB) 42.5 ± 6.3 (A-SF/P3HB + GDF-5)	7.7 ± 0.4 (SF/P3HB) 7.3 ± 0.7 (S-SF/P3HB) 1.9 ± 0.2 (A-SF/P3HB) 2.0 ± 0.1 (A-SF/P3HB + GDF-5)	—	52.7 ± 3.0 (SF/P3HB) 54.1 ± 3.2 (S-SF/P3HB) 64 ± 1 (A-SF/P3HB) 56 ± 3 (A-SF/P3HB + GDF-5)	2.33 ± 0.18 (SF/P3HB) 2.27 ± 0.16 (S-SF/P3HB) 1.0 ± 0.2 (A-SF/P3HB) 0.9 ± 0.4 (A-SF/P3HB + GDF-5)	—
Yuan et al. (2016)	Tendon	*In vitro*	Aligned Photo-crosslinked	PCL/PTMC-MA	mMSCs		∼600 nm		As-fabricated before Photo-crosslinked after Photo-crosslinked	11.16 ± 0.55 (PCL) 15.26 ± 2.37 [PCL/PTMC-MA (3:1)] 17.00 ± 1.22 [PCL/PTMC-MA (1:1)] 19 ± 3.42 [PCL/PTMC-MA (1:3)] 31.13 ± 1.30 [PCL/PTMC-MA (1:3)]	4.96 ± 0.94 (PCL) 8.35 ± 1.24 [PCL/PTMC-MA (3:1)] 10.31 ± 1.82 [PCL/PTMC-MA (1:1)] 13.21 ± 0.47 [PCL/PTMC-MA (1:3)] 23.80 ± 3.44 [PCL/PTMC-MA (1:3)]	—	77 ± 10 (PCL) 126 ± 22 [PCL/PTMC-MA (3:1)] 149 ± 12 [PCL/PTMC-MA (1:1)] 159 ± 9 [PCL/PTMC–MA (1:3)] 170 ± 22 [PCL/PTMC-MA (1:3)]		
Sensini et al. (2021a)	Tendons and Ligaments	*In vitro*	Aligned and random	Nylon 6	—	—	0.21 ± 0.05 µm (2,500 rpm) 0.23 ± 0.06 µm (1,750 rpm) 0.24 ± 0.06 µm (1,000 rpm)	—	As-fabricated	1.1 ± 0.23 (1,000 rpm) 2.7 ± 0.49 (1,750 rpm) 4.4 ± 0.78 (2,500 rpm)	—	1.9 ± 0.53 (1,000 rpm) 4.6 ± 1.2 (1,750 rpm) 6.5 ± 1.6 (2,500 rpm)	2.8 ± 0.25 (1,000 rpm) 3.4 ± 0.36 (1,750 rpm) 3.1 ± 0.37 (2,500 rpm)	—	—
Lim et al. (2021)	Tendons and ligaments	*In vitro*	Aligned	PCL/gelatin	—	—	200–800 nm	—	—	—	—	—	—	—	—
El Khatib et al. (2021)	Tendons	*In vitro*	Aligned	PLGA	oAECs	—	1.33 ± 0.17 µm	—	As-fabricated	∼400 (0 W) ∼200 (20 W)	-	∼38 (0 W) ∼17 (20 W)	∼100 (0 W) ∼10 (20 W)	—	—

The advantage of using natural polymer-based fibers is that they are comparable to natural tendon ECM (Salvatore et al., 2021), which is beneficial for cell adhesion, proliferation, and differentiation (Zha et al., 2020). However, natural fibers have low mechanical strength. Silk, on the other hand, is readily available and inexpensive, has a relatively slow controlled degradation rate, and possesses excellent mechanical properties. Numerous previous studies have demonstrated that silk is an ideal scaffold material for AT tissue engineering and has a promising clinical application (Chen et al., 2021a). Insoluble collagen fibers are more suitable for scaffold preparation than soluble collagen since their mechanical properties are closer to those of natural tendon ECM (Yang et al., 2019).

Moreover, natural polymers blended with synthetic polymers increase their mechanical strength and promote tissue regeneration (Jayasree et al., 2019; Lim et al., 2021; Liu et al., 2022). The combination of natural and synthetic polymers can maximize the benefits of each biomaterial, allowing the composite scaffold to possess significant properties (Nikolova and Chavali, 2019). Therefore, different natural/synthetic polymers, such as PCL/nCol (Jayasree et al., 2019), PCL/chitosan (Almeida et al., 2019), Col/PDS (Moshiri et al., 2013; Moshiri et al., 2015), Col/BDDGE (Sandri et al., 2016), SF/PEO (Yi et al., 2018), and SF/PCL (Yuan et al., 2016) have been developed for Achilles tendon tissue engineering (Jayasree et al., 2019; Yi et al., 2018; Moshiri et al., 2015; Yuan et al., 2016; Li et al., 2020a) (Table 1). The FDA has approved drug delivery systems and sutures for PCL. However, PCL is extremely hydrophobic, resulting in reduced cell-scaffold interactions. Blends of PCL with hydrophilic natural polymers such as cellulose acetate (CA) improve its hydrophilic, biocompatibility, and matrix erosion profile (Ramos et al., 2019).

In addition, some special materials are also used in nanoscaffolds. MOFs assembled by metal ions and organic connectors possess the advantages of large pore volume, high specific surface area, and hydrophilicity, which can enhance the performance of tissue scaffolds. In addition, these types of MOFs have great potential for tissue repair and regeneration (Chen J. et al., 2020; Luo et al., 2021; Yang et al., 2022). It has been established that motion-driven electromechanical stimulation of tendon tissue by ferroelectric nanofibers can modulate ion channels and specific tissue regeneration signaling pathways *in vitro* (Fernandez‐Yague et al., 2021).

### 4.3 Nanofibrous Scaffolds for Achilles Tendon Regeneration

Due to their excellent bionic properties, numerous studies over the past decade have established the beneficial effects of nanofibrous scaffolds as cell delivery scaffolds for tendon regeneration (Moffat et al., 2009). In addition, several studies have investigated nanofibrous scaffolds with alignment and/or porosity gradient (Li et al., 2020b), metal-organic framework (Yang et al., 2022), mineral gradient (Grue et al., 2020; Zhou et al., 2020), and growth factor gradient (Madhurakkat Perikamana et al., 2018) for tendine-bone interface healing (Lowen and Leach, 2020; He et al., 2021). Table 1 shows a variety of nanofibrous scaffolds for regeneration of the Achilles tendon and tendine-bone region.

Wang et al. (2016) demonstrated the potential of nanofiber as scaffolds for AT regeneration. Random and aligned electrospun nanofibers of PCL/PEO/gelatin were inoculated with hDFs to analyze the *in vitro* efficiency of the scaffold, and cell-free scaffolds were implanted in rat AT fractional defects to study the effects of AT regeneration. *In vitro* experiments revealed elongated cell morphology and tenogenic differentiation of seeded hDFs on aligned nanofibers and irregular morphology on the random nanofibers. In addition, elongated cells produced more parallel aligned collagen fibers ECM than irregularly shaped cells. *In vivo experiments* revealed that endogenous cells were able to infiltrate aligned nanofiber scaffolds more efficiently than random nanofiber scaffolds. In the aligned group, the regeneration tissue structure of parallel collagen fibers was superior to that of the random group, and the cell density was relatively lower. It has been demonstrated that well-aligned nanofibers can induce tendon differentiation in host fibroblasts; consequently, they could be used for *de novo* AT regeneration *via* cell-free methods together with tenogenic inductive nanofiber aligned scaffold (Wang et al., 2016). Chen et al. (2017) studied the effect of aligned PCL/SF (APSF) nanofiber scaffolds on AT regeneration *in vivo* and *in vitro*. By binding rDFBs to APSF, the gene expression of tendon marker proteins (fibronectin, Col I, and biglycan) of rDFBs was upregulated, and there was no statistical difference between the maximum load of regenerated AT tissue and that of normal AT.

Cai et al. (2018b) successfully prepared a random and ordered double-layer P (LLA-Cl) /SF nanofiber scaffold using the electrostatic spinning method to study tendon-bone regeneration in AT. After 12 weeks, the maximum breaking strength and stiffness of AT in the double-layer scaffold group were significantly higher than in the random nanofiber group and the control group. Aligned and random double-layer P (LLA-Cl) /SF nanofiber scaffolds can enhance gradient microstructure and tendine-bone bonding by improving collagen tissue maturity and organization, inducing new bone formation, and expanding fibrous cartilage area (Cai et al., 2018b).

Numerous research has investigated various strategies for guiding stem cell phenotypic expression and manipulating tendon matrix properties using different fibrous scaffolds (Guner et al., 2020; Sarıkaya and Gümüşderelioğlu, 2021; Zhu et al., 2021). The stem cells formed more mature tendinous tissue on aligned fibers and displayed greater osteogenic differentiation on random fibers (Yin et al., 2015a; Orr et al., 2015; Guner et al., 2020). The mechanical properties and self-assembly of insoluble collagen fibers are more similar to natural collagen ECM than those of soluble collagen. Yang et al. (2019) fabricated aligned and random nanofiber scaffolds of insoluble collagen for *in vitro* and *in vivo* Achilles tendon regeneration. The tensile strength of the nanofiber scaffold was comparable to that of natural rat AT. *In vitro* assays of tendon-related genes and proteins indicate that oriented scaffolds induce much more tenogenic differentiation of rBMSCs than random scaffolds by producing an elongated cell shape morphology on fibers. The rat AT repair study revealed that rBMSCs on orientated scaffolds can produce healing properties comparable to those of autologous AT. Wu et al. (2021) prepared wavy nanofiber scaffolds by adjusting the PPDO/SF ratio. *In vitro*, the combination of mechanical stimulation and GDF-5 enhanced the tenogenic differentiation of hADMSCs and maintained the viability and phenotype of hTCs. Furthermore, piezoelectric descendent electric fields generated during physiological exercise provide additional bioelectrical cues to activate tendon-specific regeneration pathways (Gouveia et al., 2017). Several studies have demonstrated that the motion-driven electromechanical stimulation of tendon tissue by piezoelectric bioelectric devices can regulate ion channels and affect specific tissue regeneration signaling pathways *in vitro* (Cheah et al., 2021; Lee et al., 2021). Fernandez‐Yague et al. (2021) studied the piezoelectric collagen nanofibrous scaffold made of arrays of nanofibers constructed from a ferroelectric material known as poly (vinylidene fluoride-co-tri-fluoroethylene) for rat AT regeneration. It is a self-powered piezoelectric material that represents a paradigm shift in biomedicine, eliminating the need for external power or batteries and complementing existing mechanical therapies to accelerate the repair process. *In vitro* experiments of mechanical and bioelectric stimulation of hTDCs revealed that BMP, MAPK, FAK, and Wnt/β-catenin form a complex signal network that controls tenogenic differentiation. Using a rat model of mechanical acute tendon rupture, the researchers demonstrated the efficacy of EMS in regulating ion channel expression (PIEZO1/2, TRPA1, and KCNK3/4) and the disrupted regeneration process associated with BMP signal overactivation. This discovery lays the engineering foundation for a variety of synthetic piezoelectric bioelectric nanofibrous scaffolds that enable bioelectrical control of the AT tissue regeneration process (Fernandez-Yague et al., 2021).

The tenogenic differentiation of stem cells in functional tendon tissue engineering relies not only on biophysical clues of scaffolds but also on biochemical clues (Theiss et al., 2015). Yuan et al. (2021) prepared highly oriented PLLA nanofiber scaffolds by electrostatic spinning; the surfaces of these scaffolds were modified with two key tendon ECM components, CS and COL1. *In vitro* tenogenic differentiation of hBMSCs using PLLA (core) /CS-COL1 (shell) fibers, revealed higher cell spreading and proliferation rates on aligned PLLA/CS-COL1 fibers compared to plain PLLA fibers. The expression of tendon-related genes COL1, SCX, and TNMD was significantly increased. Introducing mechanical stimulation had a synergistic effect on the tenogenic differentiation of hBMSCs. Higher expression of Smad3, TGF-βR2, and TGF-β2 was observed in cells on the PLLA/CS-COL1 fiber matrix, indicating that PLLA/CS-COL1 nanofibers induce tenogenic differentiation of hBMSCs by activating the TGF-β signaling pathway. The role of PLLA/CS-COL1 in promoting tissue regeneration was confirmed in a rat AT repair model (Yuan et al., 2021). Wang et al. (2021) used transcriptional profiling to compare changes in gene expression associated with tendon development in rats and discovered that Postn is a secreted ECM protein that regulates tenogenic differentiation potential and TSPCs stemness. Further, parallel ACF loaded with rPOSTN was implanted into the damaged AT of rats. The ACF-rP group was found to have a well-organized collagenous fibrous structure and a large number of spindle tendine-like cells. After 8 weeks of remodeling and healing, there was a significant reduction in inflammation. In comparison to the ACF group, the ACF-rP group achieved more satisfactory regeneration outcomes, as shown by the tighter and ordered arrangement of ECM deposits, which resembled natural tendons. In addition, it was demonstrated that TSPCs cultured with parallel collagen fibers loaded with rPOSTN had excellent spheroidization ability and the ability to regenerate tendon ECM structure. Currently, organoid and spheroid models are extensively used in stem cell culture. Therefore, this discovery may aid in the optimization of TSPCs culture techniques, and the discovery of additional genes that promote TSPCs function, AT regeneration, and repair (Wang et al., 2021). TSA is reported to explain the mechanism by which MSCs differentiate into functional hepatocellular, adipocyte, and cardiomyocyte cells (Yin et al., 2015b; Li et al., 2020c). Given the role of these epigenetic mechanisms in regulating MSCs differentiation and the observation of decreased HDACs expression in cells cultured on aligned fibers, Zhang et al. (2018) proposed a strategy to enhance the tenogenesis effect of aligned fibers using a small molecule of TSA, an HDACs inhibitor. Tendon-related gene and protein expression demonstrated that TSA-containing targeted scaffolds promoted TSPCs tendon differentiation more effectively than targeted fibers alone. Importantly, random fibers induced the tenogenic differentiation of TSPCs with extremely low efficiency, but the TSPCs tenogenic differentiation was significantly enhanced when random fibers bind to TSA. This study implies that TSA combined with the aligned nanofiber scaffolds may be a more effective strategy for promoting stem cells to tenogenic differentiation and promoting AT defect repair.

#### 4.3.1 Limitations of Nanofibrous Scaffolds

Although nanofibrous scaffolds have the advantages of bionic properties and structure similar to ECM, nanofiber scaffolds have some limitations. A number of the discussed studies call for increased mechanical properties for AT regeneration. Compared to microfiber matrices, the mechanical under construction of nanofiber scaffolds is a key limitation that must be overcome (Tong et al., 2012; Bongiovanni Abel et al., 2020). In addition, as a result of their relatively high packing density and limited thickness, these traditional nano-scaffolds have relatively poor inherent limitations of cell penetration (Han et al., 2021). The development of multilayer nanofibrous scaffolds increases cell infiltration and porosity (Rothrauff et al., 2017). Nevertheless, nanofibrous textile-based scaffolds demonstrate superior cell infiltration, distribution, and biomechanics compared to multilayer nanofibrous scaffolds (Shalumon et al., 2012; Khorshidi et al., 2016).

### 4.4 Multiscale Nanofibrous Scaffolds

**FIGURE 4:**
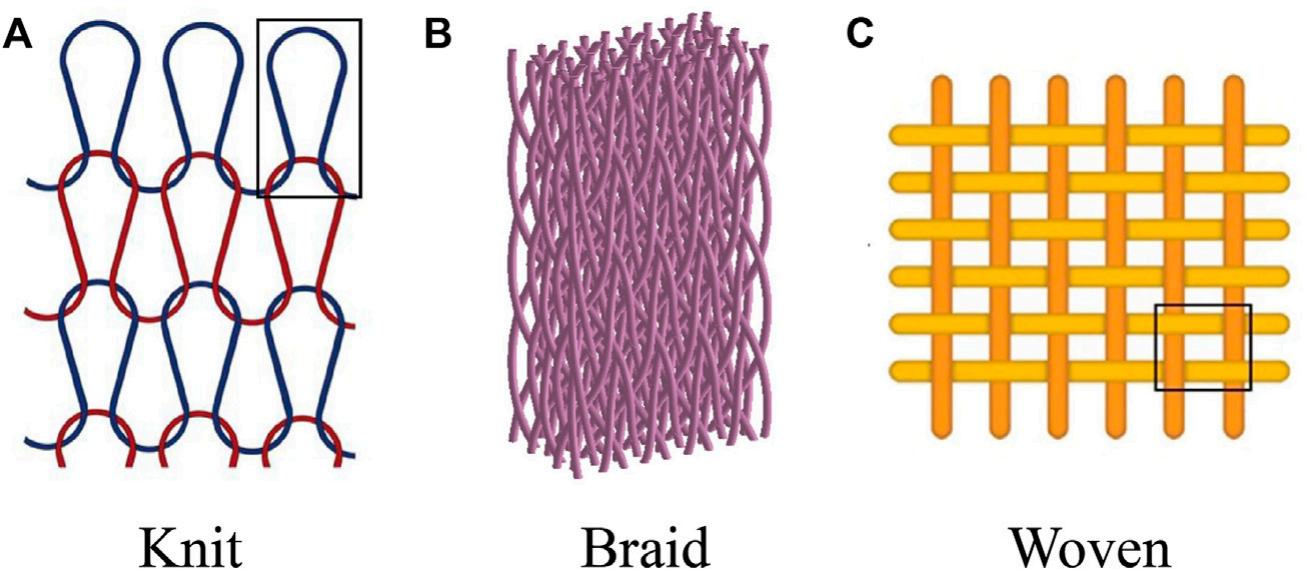
Multiscale nanofibrous textile-based scaffolds for the regeneration of AT regeneration. **(A)** Knit. Permission to reproduce was granted under the conditions of the license (CC BY 4.0). Copyright 2021, Ayodele et al. (2021) **(B)** Braid. Permission to reproduce was granted under the conditions of the license (CC BY 4.0). Copyright 2019, Ding et al. (2019) **(C)** Woven. Permission to reproduce was granted under the conditions of the license (CC BY 4.0). Copyright 2021, Yun et al. (2021).

To overcome the limitations of nanofibrous scaffolds, integrating nanofibers with different structures is an attractive research area (Liu et al., 2020; Jin et al., 2018; Chen Y. et al., 2020). It has been demonstrated that textile structures have enhanced mechanical properties and can mimic natural structures by providing a wide range of fiber forms and pore sizes (Saveh-Shemshaki et al., 2019; Ouyang et al., 2002; Chen et al., 2012). Textile-based scaffolds, such as knit, braid, and woven (Jiang et al., 2021) (Figure 4), have the potential to mimic the mechanical properties of natural tendons, overcoming the limitations of nanofibrous scaffolds, and are used in many tendon regeneration studies (Zhang et al., 2015b; Wu et al., 2017b; Cai et al., 2018a; Araque-Monrós et al., 2020; Han et al., 2021).

One strategy is to create multiscale nanofibrous textile-based scaffolds using strands or yarns of nanofibers (Almeida et al., 2019; Chen et al., 2021d; Ma et al., 2022). Another method is to coat the fabric with nanofibers (Chen L. et al., 2020; Olkhov et al., 2018). Nanofibrous strands or yarns have the potential to reconstruct the structure of natural collagen fibers (Figure1) in tendon nanofibrous to microfibrous construction (Mouthuy et al., 2015; Cai et al., 2020). Nanofibrous strands or yarns can be braided/twisted to replicate fiber bundles, or knitted/woven to simulate 3D tendon macrostructures (Li et al., 2019b; Silva et al., 2020). Studies on multiscale nanofibrous textile-based scaffolds for tendon regeneration are listed in Table 2.

**TABLE 2:** Summary of multiscale nanofiber-based tendon regeneration scaffolds.

References	Application	Animal Model	Scaffold	Material	Cell	Bioactive molecule	Morphology		Mechanical properties						
							Fiber diameter	Porosity (%)		Young’s modulus (MPa)	Ultimate stress (MPa)	Yield stress (MPa)	Ultimate stress (%)	Stiffness (N/mm)	Maximum force (N)
Jayasree et al. (2019)	Achilles tendon	*In vivo* rabbit	Braided multiscale nanofibrous scaffold	PCL/Col/alginate	rTenocytes	bFGF	300–400 nm (collagen nanofibers) 8–10 μm (Multiscale fibers composed of aligned PCL microfibers)	—	As-fabricated	—	—	89.4 ± 5.3 (braided scaffold)	—	—	46.75 ± 3.2 (braided scaffold)
Zhao et al. (2019)	Achilles tendon	*In vivo* rat	Knitted scaffolds	PLGA	rBMSCs	bFGF	15 μm (PLGA yarns)	91%	transplant after 2 weeks transplant after 8 weeks	33.76 ± 5.75 (PLGA/MSCs/bFGF) 22.46 ± 4.27 (PLGA) 16.90 ± 1.66 (control) 56.31 ± 9.64 (intact tendon) 45.73 ± 11.64 (intact tendons) 43.65 ± 5.09 (PLGA/MSCs/bFGF) 25.61 ± 5.30 (control)	—	—	—	∼70 (intact tendon) ∼25 (PLGA/MSCs/bFGF) ∼18 (control) ∼70 (intact tendon) ∼50 (PLGA/MSCs/bFGF) ∼35 (control)	132 ± 12.5 (PLGA/MSCs/bFGF) 71 ± 8.56 (PLGA/bFGF) 70.30 ± 6.65 (PLGA/MSCs) 68.00 ± 2.64 (PLGA) 64.00 ± 4.0 (control) 154.33 ± 10.3 (intact tendons) 105.57 ± 5.81 (PLGA/MSCs/bFGF) 71.27 ± 10.8 (control)
Cai et al. (2018a)	Achilles tendon	*In vivo* rabbit	Braided scaffolds	PET	rBMSCs	—	∼8 μm (PET yarns)	—	Scaffolds of seeded cell after 14 days then transplant after 6 weeks after 12 weeks		—	—	—	15.5 ± 0.9 (BMSC-PET) 15.2 ± 0.8 (PET) 30.0 ± 2.4 (BMSC-PET) 22.8 ± 2.8 (PET)	73.8 ± 5.4 (BMSC-PET) 68.6 ± 5.4 (PET) 124.5 ± 5.5 (BMSC-PET) 107.8 ± 5.3 (PET)
Morais et al. (2020)	Achilles tendon	*In vitro*	Braided scaffolds	PP/PET	—	—	—	—	As-fabricated	—	—	—	28.6 ± 2.2 (PP8YH) 33.9 ± 2.2 (PP16YH) 58.4 ± 3.3 (PPC16B8YH_S16YL) 80.3 ± 1.8 (PPC16B16YHS16YL) 16.5 ± 0.8 (PET16YHbraid) 53.9 ± 2.2 (C22B16YH_S16YL)	73.0 ± 1.1 (PPC16B8YH_S16YL) 108.6 ± 6.0 (PPC16B16YHS16YL) 25.1 ± 1.4 (PET16YHbraid) 208.0 ± 12.5 (C22B16YH_S16YL)	591.0 ± 12.1 (PP8YH) 1087.6 ± 32.4 (PP16YH) 8356.1 ± 279.7 (PPC16B8YH_S16YL) 16626.0 ± 217.0 (PPC16B16YH_S16YL) 1165.5 ± 63.4 (PET16YHbraid) 18359.7 ± 522.4 (C22B16YH_S16YL)
Yin et al. (2022)	Achilles tendon	*In vivo* rat	Multiscale	PLLA	mTSPCs	—	725 ± 127 nm (nanofibers) 2.098 ± 0.623 μm (microfibers)	—	As-fabricated	573.06 ± 38.57 (micro-nanofibers) 479.58 ± 49.16 (microfibers) 735.93 ± 97.99 (nanofibers)	20.06 ± 1.15 (micro-nanofibers) 12.91 ± 3.10 (microfibers) ∼30 (nanofibers)	—	∼125 (micro-nanofibers) ∼75 (microfibers) ∼150 (nanofibers)	—	—
Olkhov et al. (2018)	Achilles tendon	*In vivo* rat	Monofilaments surrounded by nanofiber	PHB/polyamide	Fibroblast	—	100–900 nm	—	As-fabricated	—	—	—	—	—	8–12 (nature) ∼16 (scaffolds)
Rashid et al. (2020)	Tendon	*In vivo* sheep	Aligned electrospun fibers reinforced by a woven monofilament mesh	PDO and PCL	—	—	electrospun layer: 802–1323 nm woven PDO layer: 114–134 μm	—	—	—	—	—	—	—	—
Chen et al. (2021d)	Tendon	*In vivo* rabbit	Nanoyarn scaffold	P (LLA-CL)/silk fibroin	rASCs	GDF-5	∼4 μm (Nanoyarn scaffold) ∼1 μm (nanofibers)	—	As-fabricated scaffolds of seeded cell after 3 day then transplant after 4 weeks transplant after 12 weeks	26.35 ± 8.21 (nanoyarn scaffold)	—	12.38 ± 1.35 (nanoyarn scaffold)	91.93 ± 4.35 (nanoyarn scaffold)	—	28.92 ± 13.30 (nanoyarn scaffold with ASCs) 10.09 ± 11.19 (nanoyarn scaffold w/0 ASCs) 45.04 ± 6.54 (nanoyarn scaffold with ASCs) 25.53 ± 12.76(nanoyarn scaffold w/0 ASCs)
Cai et al. (2020)	Tendon	*In vivo* rabbit	Microfiber/nanofiber yarn and knitted scaffolds	PCL/SF/PLCL	rBMSCs	—	—	—	As-fabricated scaffolds of seeded cell after 14 days then transplant after 6 moths	—	—	91.09 ± 2.50 (PCL-SF/PLCL) 67.99 ± 2.19 PCL-PCL)	—	∼110 (native tendon) ∼70 (PCL-SF/PLCL)	80.02 ± 3.48 (PCL-PCL) 77.39 ± 2.06 (PCL-SF/PLCL) 48.84 ± 2.90 (PCL) ∼200 (native tendon) ∼125 (PCL-SF/PLCL)
Shaohua Wu et al. (2020)	Tendon	*In vitro*	Nanofiber/microfiber hybrid yarns	PLGA/PLA	hADMSC	Tβ4	663.5 ± 200.8 nm (PLGA) 15 μm (PLAMicrofiber) 163.5 ± 14.6 μm (PLA MY) 172.8 ± 6.4 (PLGA/PLA HY)	—	As-fabricated	∼1300 (HY) ∼1250 (MY)	∼115 (HY) ∼100 (MY)	—	∼37 (HY) ∼38 (MY)	—	277.7 ± 9.0 cN (HY) 253.6 ± 10.7 cN (MY)
Shaohua Wu et al. (2017)	Tendon	*In vitro*	random and aligned nanofiber-yarn woven biotextiles	PCL/PLA	hADMSC/human tenocytes /hUVECs	—	452.3 ± 218.4 nm (aligned PCL) 484.8 ± 345.7 nm (random PCL) 460.2 ± 213.0 nm (PCL in weave) 208.5 ± 13.7 um (PCL yarn) 15 um (PLA yarn)	—	As-fabricated	∼60 (woven fabrics) ∼12 (aligned PCL) ∼4 (random PCL)	∼10 (woven fabrics) ∼5 (aligned PCL) ∼2 (random PCL)	—	∼45 (woven fabrics) ∼40 (aligned PCL) ∼240 (random PCL)	—	—
Li et al. (2019b)	Tendon	*In vitro*	Helical nanofiber yarn scaffold	PLGA/PU/PVDF-HFP/cellulose	MSCs/rMEF	—	—	—	As-fabricated	—	—	88.7 (hierarchical helix scaffold) 88.6 (primary yarn)	1,420 (hierarchical helix scaffold) 174% (primary yarn)	—	—
Sankar et al. (2021)	Tendon	*In vitro*	Electrospinning multiscale fibers	PCL/collagen	hMSCs	Plasma	193 ± 72 nm (collagennanofibers) 5.5 ± 1.5 μm (Aligned PCL microfibers) 8.8 ± 0.6 μm (Random PCL microfibers)	—	—	—	—	—	—	—	—
Xing Li et al. (2020)	Tendon	*In vitro*	Yarns nanofibers hierarchical scaffolds	PCL/CHT/CNCs/TROPO/PDA	hASCs	—	149 ± 16 μm (yarns) 134 ± 28 nm (uncoated) 144 ± 32 nm (PDA-coated) 153 ± 33 nm (TROPO/PDA-coated)	—	As-fabricated scaffolds of seeded cell after 21 days	9.0 ± 4.8 (uncoated) 4.7 ± 3.0 (PDA-coated) 5.6 ± 3.6 (TROPO/PDA-coated) 19.4 ± 12.5 kPa (uncoated) 11.5 ± 6.9 kPa (PDA-coated) 9.8 ± 5.9 kPa (TROPO/PDA-coated)	—	—	—	—	—
Araque-Monrós et al. (2020)	Tendon and ligament	*In vitro*	Braid scaffolds	PLA/HA/PLLA	Mouse fibroblasts	—	33 ± 5 μm (coating layer in dry) 270 μm (coating layer in swollen) 110 ± 80 μm (PLLA microspheres) 20 ± 14 μm (HA microspheres)	—	—	—	—	—	—	—	—
Chen et al. (2012)	Ligament	*In vitro*	Knitted Microfiber coated with a nanofibrous mesh	Silk/RADA	rBMSCs	—	10–20 nm (RADA nanofibers)	—	Scaffolds of seeded cell after 21 days	—	—	—	—	—	37.9 ± 9.2 (BMSC-seeded scaffold IV) 35.5 ± 2.5 (BMSC-seeded scaffold II) 31.3 ± 4.2 (unseeded scaffold IV)
Ma et al. (2022)	Transition/tendon	*In vitro*	Aligned nanowires in alginate hydrogel	CS/alginate	rBMSCs/rTPSCs	—	∼30 nm (CS nanowires)	—	As-fabricated	∼120 (Alg) ∼310 (20CS-S)		∼5 (Alg) ∼17 (20CS-S)	—	—	

**FIGURE 5:**
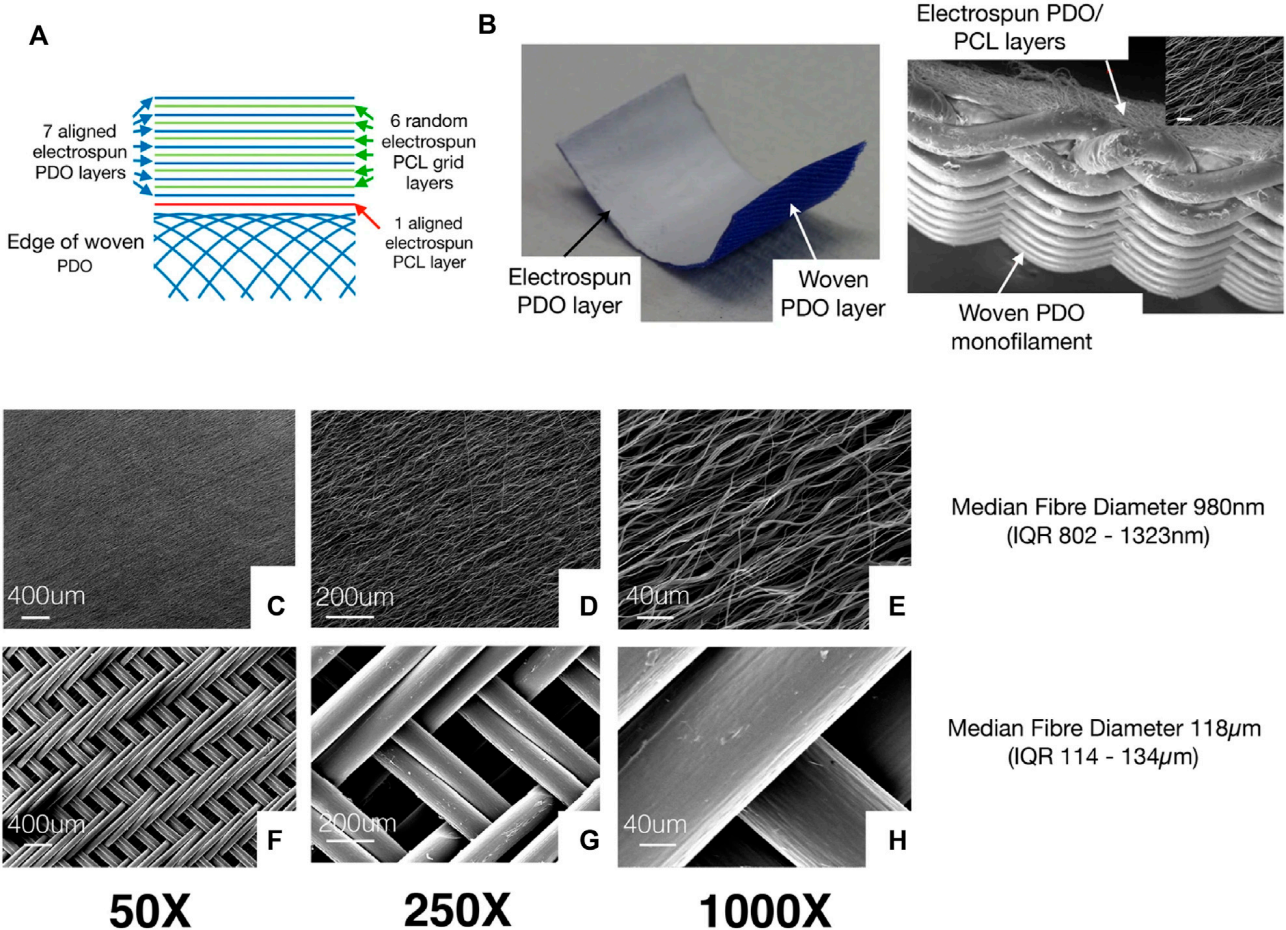
**(A)** Figure shows the different layers in the electrospinning patch. The electrospinning assembly of the patch consists of seven layers of aligned electrospun PDO fibers sandwiched alternately between 6 layers of thin PCL electrospinning mesh (used as a binder). A single layer of aligned electrospinning PCL fibers is used to join the electrospun components with a braided PDO monofilament layer. **(B)** Patch sample photo displays the white electrospun PDO layer (facing the tendon) and the blue woven PDO layer. **(C–E)** Typical SEM images showing the electrospinning layer. **(F–H)** Figures show woven patch layers at various magnifications. Permission to reproduce was granted under the conditions of the license (CC BY 4.0). Copyright 2020, Rashid et al. (2020).

Rashid et al. (2020), examined the biological potential of woven and electrospun PDO/PCL patches to induce repair responses as shown in Figure 5. The electrospinning assembly of the patch consists of seven layers of aligned electrospun PDO fibers sandwiched between six layers of thin PCL random electrospun mesh (used as a binder) (Figure 5A). A single layer of aligned electrospun PCL fibers connects the electrospinning components with a braided PDO monofilament layer (Figure 5B). All tendon defects had healed 3 months following the implantation of patch scaffolds. No residual polymer of the electrospun scaffold was found on the tendon surface. HE staining revealed various key characteristics. Electrospinning scaffolds exist in the area of cell proliferation and vascularization. The deepest layer of the section corresponds to the native lamb tendon below the patch. These areas resemble normal tendons. The serology and hematology of inflammatory markers were normal 3 months after scaffold implantation, with no indications of increased local or systemic inflammation.

Morais et al. (2020) developed various AT regeneration structures based on PET or PP multifilament yarns. These structures are based on a dual shell/core system in which the core is comprised of numerous sub-components (braided fabric) and the shell is based on the braided yarn that surrounds the core. The PET-based yarn structure exhibited a non-linear force and strain curve comparable to that of typical natural AT, showing optimal load, strain-failure, and stiffness levels for AT replacement. In addition, the PET rope also demonstrated adequate creep resistance and very promising fatigue properties in its final application, which is consistent with the native AT’s report. Jayasree et al. (2019) fabricated a braided multiscale fiber scaffold consisting of neatly arranged PCL microfibers/collagen bFGF nanofibers (mPCL-nCol-bFGF) and coated with sodium alginate to inhibit perinetendinous adhesions. Rabbit Tenocytes exhibited the highest levels of tenogenic marker expression and cellular proliferation in response to *In vitro* dynamic stimulation. AT tissue regenerated 12 weeks following surgical implantation, and the collagen morphology was neatly arranged.

The effects of multiscale structure and polymer type on cell function (after implantation of rBMSCs) and AT mechanical properties were investigated (Sankar et al., 2021; Yin et al., 2022). Zhao et al. (2019) added an exogenous BFGF-loaded fibrin gel to a porous network of knitted PLGA scaffolds. The scaffold stimulated rBMSCs proliferation and tenocytes differentiation, which synergistically enhanced the reconstruction of damaged Achilles tendons. The biomechanical test 8 weeks following transplantation revealed that the elastic modulus of the regenerated AT was comparable to that of an intact tendon (43.65 ± 5.09 MPa, 45.73 ± 11.64 MPa, respectively). Cai et al. used braided PET scaffolds to induce rBMSCs tenocytes differentiation in the absence of a bioactive molecule; the mechanical properties of regenerative AT with cellular scaffolds were significantly better than those with cell-free scaffolds.

The promising outcomes of these experiments on tendon repair scaffolds suggest a new direction for AT regeneration. Further research using multiscale nanofibrous scaffolds may contribute to the regeneration and integration of the AT structure.

## 5 Conclusion and Future Expectation

Extensive research has been conducted on AT regeneration engineering, including bioactive molecules, cell therapies, and advanced biological scaffolds such as nanofibrous scaffolds, over the past few decades. Numerous studies have demonstrated promising results for strategies aimed at bioenhanced AT repair. Nanofibrous scaffolds can mimic natural tendon structures and have the potential to provide physiological characteristics and bionic machinery. The mechanical properties and pore size limitations of nanofibrous scaffolds have prompted the development of multiscale nanofibrous scaffolds. Studies have demonstrated that these multiscale nanofibrous scaffolds promote AT regeneration.

Numerous *in vitro* or animal model researches demonstrate that AT has an excellent healing effect; however, clinical trials on humans have not been conducted, and the efficacy and safety of human application are uncertain. Therefore, future clinical studies should explore the feasibility of a strategy for human AT regeneration. Moreover, other nanomaterials and technologies suitable for AT tissue engineering need to be developed. The success of the AT regeneration project will significantly reduce the medical burden and improve the quality of people’s life.

## Glossary

AT: Achilles tendon
ECM: Extracellular matrix
hTDCs: Human tendon–derived cells
TPSCs: Tendon progenitor/stem cells
hTPSCs: Human tendon progenitor/stem cells
SCXA: Scleraxis transcription factor
MSCs: Mesenchymal stem cells
hBMSCs: Human bone marrow mesenchymal stem cells
BMSCs: Bone marrow mesenchymal stem cells
rBMSCs: Rat Bone marrow mesenchymal stem cells
ADSCs: Adipose-derived mesenchymal stem cells
hADSCs: Human adipose–derived mesenchymal stem cells
hDFs: Human dermal fibroblasts
hTCs: Human tenocytes
rDFBs: Rabbit dermal fibroblasts
hPDLSCs: Human periodontal ligament stem cells
C3H10T1/2: Mouse embryonic mesenchymal stem cell line
EVs: Extracellular vesicles
IPSCs: Induced pluripotent stem cells
AECs: Alveolar epithelial cells
siRNA: Interfering RNA
RADA: Acids arginine, alanine, and aspartic acid (Ac-(RADA)4-CONH2)
RGD: Arginine, glycine and aspartate
PDGF-BB: Platelet-derived growth factors BB
IGF-1: Insulin-like growth factor 1
bFGF: Basic fibroblast growth factor
BMP-7/12: Bone morphogenetic protein 7/12
TGF-β1/β3: Tansforming growth factor β1/β3
TGF-βR2: TGF-β receptor 2
VEGF: Vascular endothelial–derived growth factor
SDF-1α: Stromal cell–derived factor-1α
PRF: Platelet-rich fibrin
LP-PRP: Leucocyte-poor PRP
LR-PRP: Leucocyte-rich PRP
FDA: Food and Drug Administration
PCL: Polycaprolactone
PLA: Polylactic acid
PLGA: Polylactic acid-glycolic acid
PET: Polyethylene terephthalate
PEO: Poly ethylene oxide
PGA: Poly-gamma-l-glutamic acid
PP: Polypropylene
PHB: Poly-3-hydroxybutyrate
HA: Hydroxyapatite
Col: Collagen
nCol: Nanostructured collagen
COL1: collagen Type 1
COL1A1: Collagen type I alpha1
COL3A1: Collagen type III alpha1
DCN: Decorin
sGAG: Sulfated glycosaminoglycan
PDS: Polydioxanone
BDDGE: Butanediol diglycidyl ether
SF: Silk fibroin
PLLA: Poly-L-lactic acid
CS: Chondroitin sulfate
APSF: Aligned PCL/SF
ALG: Alginate
SCX: Scleraxis
SCX-GFP: Scleraxis-green fluorescent protein
TNMD: Tenodmodulin
Smad3: drosophila mothers against decapentaplegic 3
GDF5: Growth differentiation factor 5
HDACs: Histone deacetylase
TSA: Traostatin A
Postn: Periostin
rPOSTN: Recombinant periostin
ACF: Aligned collagen Fibrils
ACF-rp: ACF- rPOSTN
Oct4: Octamer-binding transcription factor 4
Sox2: Sry-related box 2
CD146: Cluster of Differentiation 146
**Ki67** (MKI67): A nuclear protein that is associated with cellular proliferation.
TEND: Electrospun tissue engineering device
PDLLA: Poly (D, L-lactide)
DMSO: Dimethyl sulphone
MAPK: Mitogen-activated protein kinase
FAK: Focal adhesion kinase
Wnt/β-catenin: pathways are known to regulate both physiological and pathological processes
EMS: Electromechanical stimulation
PIEZO1/2: Member of the mechanical gated cation channel family
TRPA1: Transient receptor potential ankyrin 1
KCNK3/4: Potassium channel subfamily K member 3
ZnO: Zinc oxide
ATDC5: Murine chondrogenic cell line
MC3T3-E1: Mouse osteoblastic cell line
MOFs: Metal-organic scaffolds
ZIF-11: Metal-organic frameworks 11
HKUST-1 (MOF-199): Hong Kong University of Science and Technology 1, is a material in the category of metal-organic frameworks
TEM: Transmission electron microscope
SEM: Scanning electron microscopy
MLT: Melatonin
Tβ4: Thymosin beta4
CC BY 4.0: Creative Commons Attribution 4.0 International
